# Enzymatic and Biological Characterization of Novel Sirtuin Modulators against Cancer

**DOI:** 10.3390/ijms20225654

**Published:** 2019-11-12

**Authors:** Vincenzo Carafa, Angelita Poziello, Laura Della Torre, Pia Giovannelli, Marzia Di Donato, Elham Safadeh, Zhijun Yu, Alfonso Baldi, Gabriella Castoria, Daniela Tomaselli, Antonello Mai, Dante Rotili, Angela Nebbioso, Lucia Altucci

**Affiliations:** 1 Dipartimento di Medicina di Precisione, Università degli Studi della Campania “Luigi Vanvitelli”, 80138 Napoli, Italy; vincenzo.carafa@unicampania.it (V.C.); angelita.poziello@unicampania.it (A.P.); laura.dellatorre@unicampania.it (L.D.T.); pia.giovannelli@unicampania.it (P.G.); marzia.didonato@unicampania.it (M.D.D.); elham.safadeh@unicampania.it (E.S.); zhijun.yu@unicampania.it (Z.Y.); gabriella.castoria@unicampania.it (G.C.); 2 Dipartimento di Scienze e Tecnologie Ambientali Biologiche e Farmaceutiche Università degli Studi della Campania “Luigi Vanvitelli”, 81100 Caserta, Italy; alfonso.baldi@unicampania.it; 3 Dipartimento di Chimica e Tecnologie del Farmaco “Sapienza” Università di Roma, 00185 Roma, Italy; daniela.tomaselli@uniroma1.it

**Keywords:** sirtuins, cancer, cell cycle, migration

## Abstract

Sirtuins, a family of nicotinamide adenine dinucleotide (NAD^+^)-dependent lysine deacetylases, are promising targets for anticancer treatment. Recently, we characterized a novel pan-sirtuin (SIRT) inhibitor, MC2494, displaying antiproliferative effects and able to induce death pathways in several human cancer cell lines and decrease tumor growth in vivo. Based on the chemical scaffold of MC2494, and by applying a structure–activity relationship approach, we developed a small library of derivative compounds and extensively analyzed their enzymatic action at cellular level as well as their ability to induce cell death. We also investigated the effect of MC2494 on regulation of cell cycle progression in different cancer cell lines. Our investigations indicated that chemical substitutions applied to MC2494 scaffold did not confer higher efficacy in terms of biological activity and SIRT1 inhibition, but carbethoxy-containing derivatives showed higher SIRT2 specificity. The carbethoxy derivative of MC2494 and its 2-methyl analog displayed the strongest enzymatic activity. Applied chemical modifications improved the enzymatic selectivity of these SIRT inhibitors. Additionally, the observed activity of MC2494 via cell cycle and apoptotic regulation and inhibition of cell migration supports the potential role of SIRTs as targets in tumorigenesis and makes SIRT-targeting molecules good candidates for novel pharmacological approaches in personalized medicine.

## 1. Introduction

It is now widely accepted that cancer displays both genomic and epigenomic deregulations [1,2]. Histone modifications, such as acetylation, play a key role in regulating gene expression and chromatin accessibility. The correct balance between histone deacetylases (HDACs) and histone acetyltransferases (HATs) is fundamental in determining and maintaining chromatin structure, and contributes to the control of different biological pathways. The best-known epigenetic enzymes targeted in cancer therapy are HDACs, which modulate the expression and activity of many proteins deregulated in cancer [3,4,5]. Several class I/II/IV HDAC inhibitors are currently being tested in phase I, II and III clinical trials [6,7,8], and some have entered the clinic. Unlike other HDACs, class III HDACs, better known as sirtuins (SIRTs) include seven proteins (SIRT1–7) [9] that share homology with the yeast deacetylase Sir2, and vary in sequence and length of both their N- and C-terminal domains [10]. SIRTs are expressed from bacteria to humans [11] and target both histone and non-histone proteins [12]. Although their two best characterized enzymatic activities are nicotinamide adenine dinucleotide (NAD^+^)-dependent lysine deacetylation and ADP ribosylation, SIRTs also catalyze other enzymatic reactions by removing other acyl groups, such as succinyl, malonyl, glutaryl, and long-chain fatty acyl groups [13,14]. Through modulation of different cellular pathways such as DNA repair, transcriptional regulation, metabolism, proliferation, death, aging, and senescence, SIRTs regulate cancer cell proliferation, progression, and death, suggesting their potential use as targets in cancer therapy. To date, the physiological SIRT inhibitor (SIRTi) nicotinamide (NAM) [15] and several other structurally unrelated SIRTi have been reported [16,17]. Although few SIRTi have been well characterized, some of these compounds such as EX527 [18,19], cambinol [20,21], salermide [22,23], UVI5008 [24,25], and tenovins [26,27] exhibit potential anticancer activity in specific contexts. Recently, we identified a novel pan-SIRTi, MC2494, with enhanced tumor-selective potential in vitro, ex vivo, and in vivo in both xenograft and allograft cancer models [28]. In addition, we investigated its anticancer activity by characterizing the type of cell death mechanism activated, and observed a novel kind of tumor-selective apoptosis. This proposed activity of MC2494 corroborates the role of SIRT1 in tumorigenesis and prompted us to further explore the activity of MC2494 and to design new small MC2494-derived molecules able to modulate SIRT activity. We also evaluated the action of MC2494 on cell cycle progression and migration, characterizing some of its derivatives at biological and enzymatic levels.

## 2. Results

### 2.1. MC2494-Related Derivatives and Their Enzymatic Characterization

**Figure 1:**
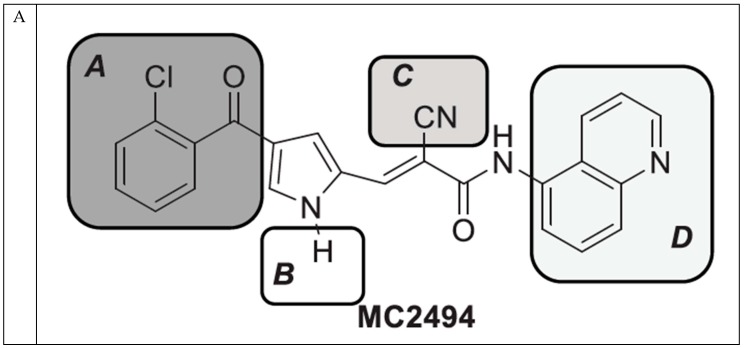

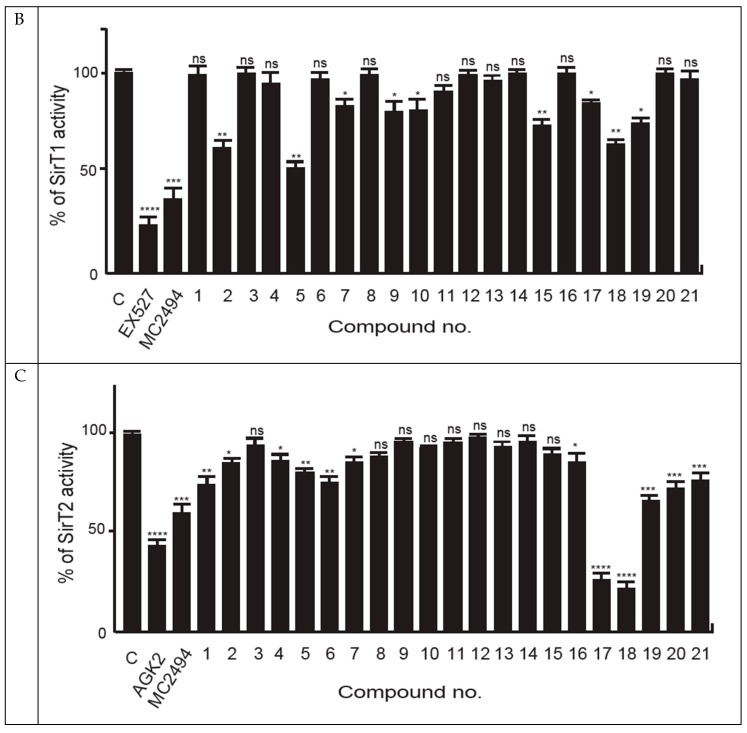

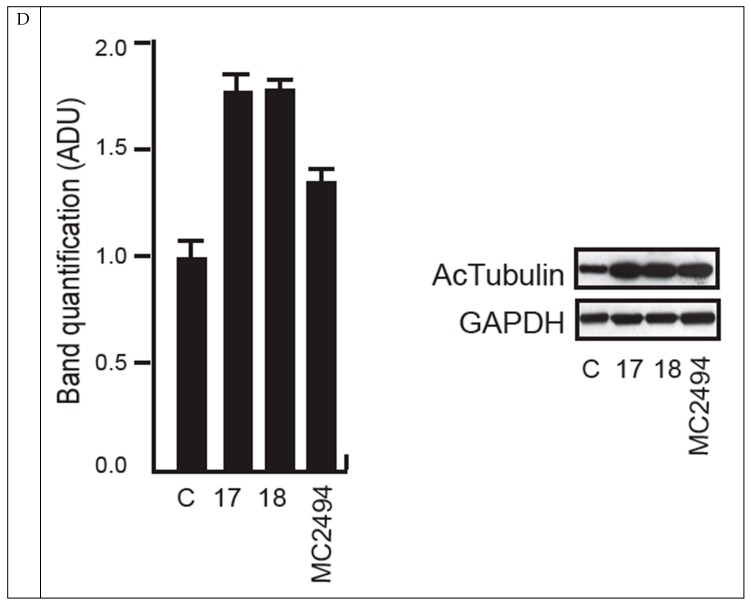
Enzymatic evaluation of novel MC2494-related derivatives. (**A)** The four different portions in MC2494 structure subjected to chemical modifications. (**B**) Sirtuin1 (SIRT1) enzymatic assay carried out with the investigated compounds (50 μM). MC2494 (50 μM) and EX527 (5 μM) were used as controls. (**C**) SIRT2 enzymatic assay carried out with the investigated compounds (50 μM). MC2494 (50 μM) and AGK2 (50 μM) were used as controls. Graph shows the mean of three independent experiments with error bars indicating standard deviation. (**D**) Western blot analysis of acetyl tubulin. Glyceraldehyde 3-phosphate dehydrogenase (GAPDH) was used as control for equal loading. Western blots were normalized through densitometry analysis, performed using the Image J Gel Analysis tool. **** *p*-value ≤ 0.0001, *** *p*-value ≤ 0.001, ** *p*-value ≤ 0.01, * *p*-value ≤ 0.05, ns *p*-value > 0.05 vs. control cells.

In order to optimize the potency of 3-(4-(2-chlorobenzoyl)-1H-pyrrol-2-yl)-2-cyano-N-(quinolin-5-yl) acrylamide (the MC2494 prototype) and to determine at least preliminary structure–activity relationships (SARs), we identified four different portions (A–D) in the structure of MC2494 and subjected them to a systematic chemical modification (Figure 1A and Table 1). Specifically, the 2-chlorobenzoyl group at position 4 of the pyrrole ring (portion A) was removed, substituted with 3- and 4-chlorobenzoyl moieties, left unsubstituted or changed to differently decorated (methyl, methoxy or fluoro) benzoyl moieties; the hydrogen at position 1 of the pyrrole ring (portion B) was substituted with a methyl group, while the cyano moiety at position 2 of the acrylamide chain (portion C) was changed to a carbethoxy group; lastly, the 5-quinolinyl substituent at the amide N of the acrylamide chain (portion D) was changed to different monocyclic and bicyclic (hetero)aromatic rings. Using this chemical approach, we identified 21 MC2494-related compounds (Table 1 and Supplementary Table S1). The effect of MC2494 derivatives on SIRT1 and SIRT2 protein expression was then investigated in leukemia U937 cells by Western blot. MC2494 and compounds 7 and 18 markedly decreased the expression of SIRT1 (Supplementary Figure S1A). A robust decrease in SIRT1 protein expression was also observed with compounds 1, 4, 5, 16, and 20. An analysis of SIRT2 protein expression revealed that compounds 7, 18, and 19 robustly decreased the expression of SIRT2 (Supplementary Figure S1B). A considerable decrease in SIRT2 protein expression was also observed with several other compounds (compounds 4, 7, 16, 17, and 20) (Supplementary Figure S1B). To clarify whether these compounds also exerted an inhibitory effect against SIRT1 and SIRT2, a fluorescent enzymatic assay of the compounds was performed at a concentration of 50 μM against SIRT human recombinant enzymes produced in *Escherichia coli*. Together with MC2494, EX527 and AGK2 were included as controls due to their well-known inhibitory activities (EX527 for SIRT1 and AGK2 for SIRT2). Although a decrease in protein expression was detected, only few compounds displayed inhibition against the two SIRTs. Three of the tested MC2494 analogs (compounds 2, 5, and 18) retained an inhibitory activity against SIRT1 (40%, 50%, and 35%, respectively), although less potent than the prototype (Figure 1B). Regarding the effect against SIRT2, compounds 17 and 18 markedly decreased SIRT2 enzymatic activity, showing a higher inhibitory effect (74% and 79%, respectively) than that exerted by MC2494, while compound 19 displayed a weak SIRT2 inhibition (34%), comparable to that of the prototype (Figure 1C). To corroborate the results of the enzymatic assay, the functional activity of the compounds was studied by Western blot of histone H3 acetylated at lysine K9/14 (H3K9/14ac) and acetyl tubulin, well-known targets of SIRT1 and SIRT2 deacetylation, respectively. Following treatment with the MC2494 derivatives, no variation in H3K9/14ac was observed (Supplementary Figure S1C), while Western blot of acetyl tubulin showed a strong induction, confirming the functional inhibitory activity of compounds 17 and 18 toward SIRT2 (Figure 1D). Among all modifications performed on the benzoyl moiety (portion A) of MC2494 prototype, only the shift of the chloro substituent from ortho to meta position (compound 5) retained a good inhibitory effect against SIRT1 (50%), although less potent than the prototype, with a weak effect on SIRT2 (20%). The removal of the entire benzoyl group, the elimination of chloro at the 2-benzoyl position as well as its substitution with both electron-withdrawing (fluoro) and -donating (methyl, methoxy) groups always led to a decrease in activity against both SIRT1 and SIRT2 (compare 2, 4, 7, 9, and 11 with MC2494). As seen for chloro derivatives, independent of the benzoyl substituent, the shift from ortho to meta position almost always resulted in a further drop in potency against both SIRT1 and SIRT2 (compare 7 with 8, 9 with 10, 11 with 12). Methylation of the N1 of the pyrrole ring (portion B) as well as substitution of the 5-quinolinyl ring at the amide N of the acrylamide chain (portion D) with a 1-naphthyl, 2-pyrimidinyl, and 4-pyridyl ring, independently of the decoration at portion A, led to a significant drop in SIRT1 and SIRT2 inhibiting activity with the only partial exception of the 2-pyrimidinyl analog (compound 15) of MC2494, which still retained some SIRT1 inhibitory activity (28%). Interestingly, substitution of the cyano moiety at position 2 of the acrylamide chain (portion C) with a carbethoxy group resulted in a significant change in the selectivity profile of the unselective MC2494 prototype. Both the carbethoxy derivative of MC2494 (compound 17) and its 2-methyl analog (compound 18) were, in fact, significantly less potent against SIRT1 and more effective against SIRT2 than MC2494. Together, our findings show that all the carbethoxy-containing derivatives (compounds 16–21), regardless of the decoration at portions A and D, resulted more potent against SIRT2 than SIRT1, suggesting that this substituent may have a crucial role in discriminating between these two proteins.

### 2.2. Effects of MC2494 Derivatives on Cell Cycle Progression

The biological effect of the new hypothetical modulators on cell cycle progression and cell death induction was studied via flow cytometry by fluorescence-activated cell sorting (FACS). To better discriminate their effects, FACS analysis was performed in different human cancer cell lines: leukemia U937 cells, triple-negative breast cancer MDA-MB-231 cells, and embryonic kidney cancer HEK293T cells. Each cell line was treated with all MC2494 analogs for 24 h at a final concentration of 50 μM, and their action was compared only to MC2494, used as a control, since EX527 is not able to induce variation in cell cycle progression and in cell death induction (Supplementary Figure S2).

**Figure 2:**
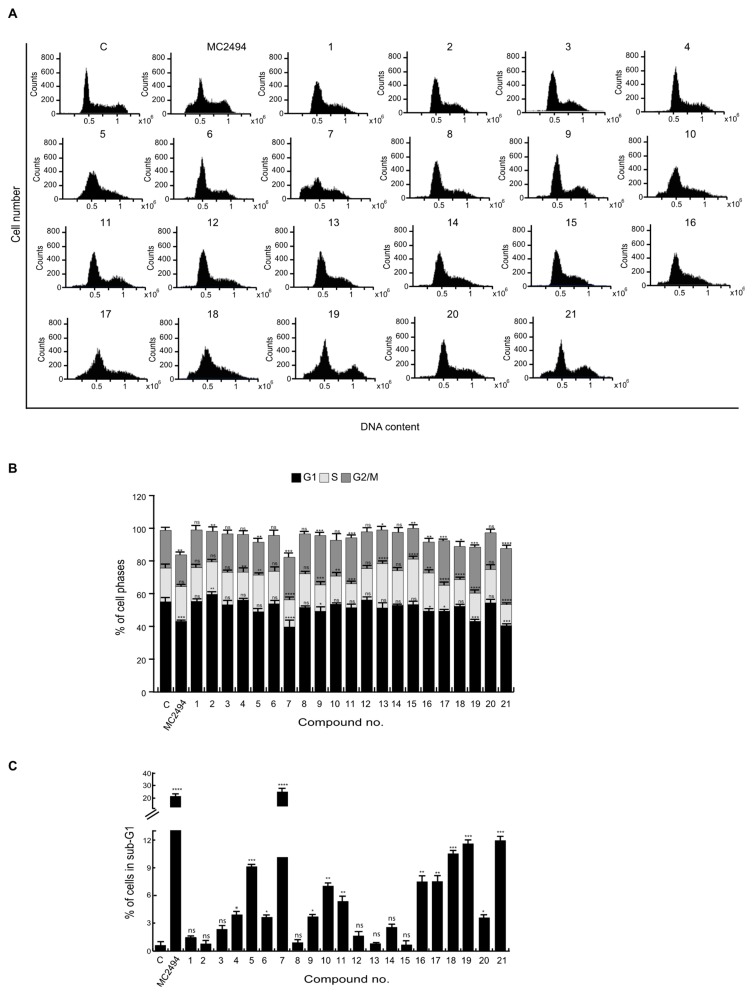
Effects of novel MC2494-related derivatives on cell cycle regulation. U937 cells were treated with the compounds at 50 μM concentration for 24 h. (**A**) Graphs of cell cycle distribution, (**B**) cell cycle analysis, (**C**) sub-G1 analysis. Graphs show the mean of at least three independent experiments with error bars indicating standard deviation. Values are mean ± standard deviation (SD) of biological triplicates. **** *p*-value ≤ 0.0001, *** *p*-value ≤ 0.001, ** *p*-value ≤ 0.01, * *p*-value ≤ 0.05, ns *p*-value > 0.05 vs. control cells.

In U937 cells, we observed a slight decrease in the percentage of cells in G1 and S phases, and a slight increase in G2/M phase following treatment with compound 19 (G1, 43.2%; S, 17.3%), compounds 17 and 18 (S, 15.8% and 16.4%, respectively), and compound 9 (G2/M, 30.2%). In contrast, compound 7 determined a strong decrease in G1 and S phases (G1, 34.1%; S, 11.1%), while treatment with compound 21 led to a strong decrease in S phase and an increase in G2/M phase (S, 12.9%; G2/M, 34.3%) (Figure 2A,B).

The effects observed on cell cycle progression in MDA-MB-231 and HEK293T cancer cell lines were less evident (Supplementary Figure S3A,B). In MDA-MB-231 cells, compounds 7, 14, and 16 determined a slight variation in cell cycle progression. Specifically, treatment with compound 7 led to a decrease in G1 phase and a slight increase in G2/M phase compared to control cells (G1, 49.4%; G2/M, 26.5%). Treatment with compound 14 led to a decrease in G1 phase and a slight increase in S phase (G1, 47.4%; S, 30.3%), while treatment with compound 16 determined only a slight increase in G2/M phase (G2/M, 26.5%). Interestingly, after induction with compound 19 and 21, a strong variation in cell cycle progression was observed. Compound 19 induced a strong decrease in G1 phase and an increase in G2/M phase (G1, 30.2%; G2/M, 44.1%). Induction with compound 21 led to a decrease in G1 phase and an increase in S phase (G1, 39.8%; S, 35.6%) (Supplementary Figure S3A). In HEK293T cells, compounds 6, 11, 14, and 17 induced a reduction in S phase (13.8%, 14.6%, 16.5%, and 15.1%, respectively). Compounds 18 determined a strong decrease in G1(18, 38.4%) and compound 17 in G2/M (18.2%) phases (Supplementary Figure S3B).

### 2.3. Analysis of MC2494 Analogs on Cell Death Regulation in Cancer

We previously demonstrated that MC2494 strongly induces cancer-selective cell death [28]. Therefore, we investigated the effects of MC2494 derivatives on cell viability and cell death induction. We performed FACS analysis of hypodiploid sub-G1 peak on fixed cells and propidium iodide (PI) incorporation on live cells to evaluate DNA fragmentation and dead-cell membrane permeabilization, respectively. Each cell line was treated with all MC2494 analogs for 24 h at a final concentration of 50 μM, and their action was compared to that of MC2494, used as a control. After 24 h of treatment in U937 cells, only compound 7 led to a strong increase in sub-G1 peak (25%), displaying a higher effect than MC2494. With compounds 18, 19, and 21, a slight effect on DNA fragmentation was detected; this effect was lower compared to MC2494 (11%, 11.6%, and 12.2%, respectively vs. 20%) (Figure 2C). In HEK293T cells, a very slight induction in DNA fragmentation was observed after treatment only with compounds 17, 18, and 19 (7.5%, 9%, and 8.4%, respectively) (Supplementary Figure S4A, left panel). No appreciable effect was observed in MDA-MB-231 cells; only compound 18 led to a slight increase (7.8%) (Supplementary Figure S4B, left panel), suggesting that this cell line might be more resistant.

**Figure 3:**
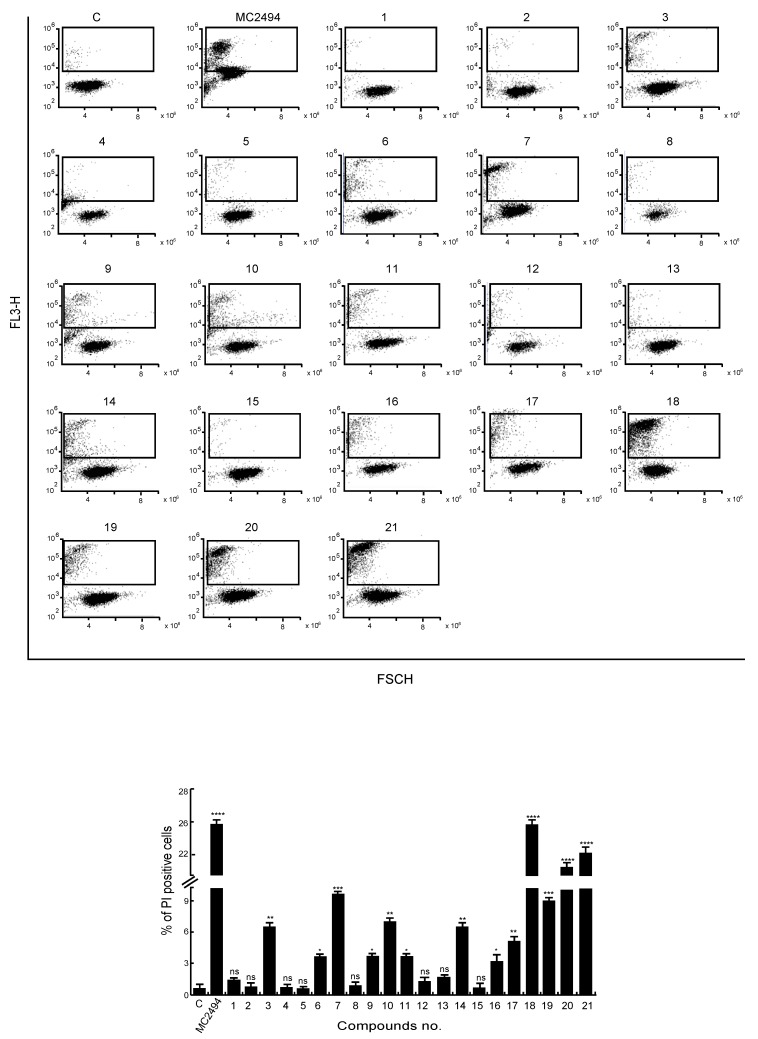
Effects of novel MC2494-related derivatives on propidium iodide (PI) incorporation. U937 cells were treated with the compounds at 50 μM concentration for 24 h. (top panel) Graphs of PI distribution, (bottom panel) PI evaluation. Graphs show the mean of at least three independent experiments with error bars indicating standard deviation. Values are mean ± SD of biological triplicates. **** *p*-value ≤ 0.0001, *** *p*-value ≤ 0.001, ** *p*-value ≤ 0.01, * *p*-value ≤ 0.05, ns *p*-value > 0.05 vs. control cells.

The comparative study of cell-membrane permeabilization revealed that a lower PI incorporation compared to MC2494 was induced by compound 5 (7.7% in HEK293T), compounds 9 and 10 (7% and 7.8%, respectively in HEK293T), compound 11 (9.7% in MDA-MB-231), compound 13 (8.2% in HEK293T), compound 17 (7.4% in MDA-MB-231), compound 19 (8.7% in U937, 9.1% in MDA-MB231, 9.8% in HEK293T), and compound 20 (7.9% in HEK293T). A greater effect was observed with compound 2 (13.6% in HEK293T), compound 7 (10.3% in U937), compound 11 (15.4% in HEK293T), compound 16 (14% in HEK293T), compound 17 (13% in HEK293T), compound 18 (26.7% in U937, 11.6% in HEK293T), compound 20 (17.5% in U937), and compound 21 (22.8% in U937, 14% in HEK293T) (Figure 3 and Supplementary Figure S4A,B right panels). To characterize cell death observed after treatment with some compounds, the activation of caspase 3/7 was evaluated. Although with a lower effect compared to MC2494, compounds 7, 18, 19, and 21 were able to activate caspase 3/7 (Supplementary Figure S4C). To corroborate this result, early and late apoptotic cells were identified through double staining with Annexin V/PI. U937 cells were treated with all MC2494 analogs, and a strong effect, comparable to that of MC2494, was observed after induction with compounds 18, 20, and 21. (Supplementary Figure S5A,B). Taken together, these biological findings indicate that the chemical substitutions applied to develop MC2494-related derivatives did not confer higher efficacy in terms of cell death induction.

### 2.4. MC2494 Displays Context-Specific Regulation in Cancer Cell Cycle Progression

MC2494 blocks cell proliferation by inducing cell death in a broad panel of cancer cells and normal or immortalized non-cancer cells. In order to identify context-specific actions against cancer, we studied its effects on cell cycle distribution in human breast cancer MCF7, triple-negative MDA-MB-231, T47D, and MDA 453 cells, in human acute myeloid leukemia U937 (M5) and HL60 (M2) cells, and in a K562 cell line derived from a chronic myeloid leukemia patient expressing B3A2 bcr-abl hybrid gene.

**Figure 4:**
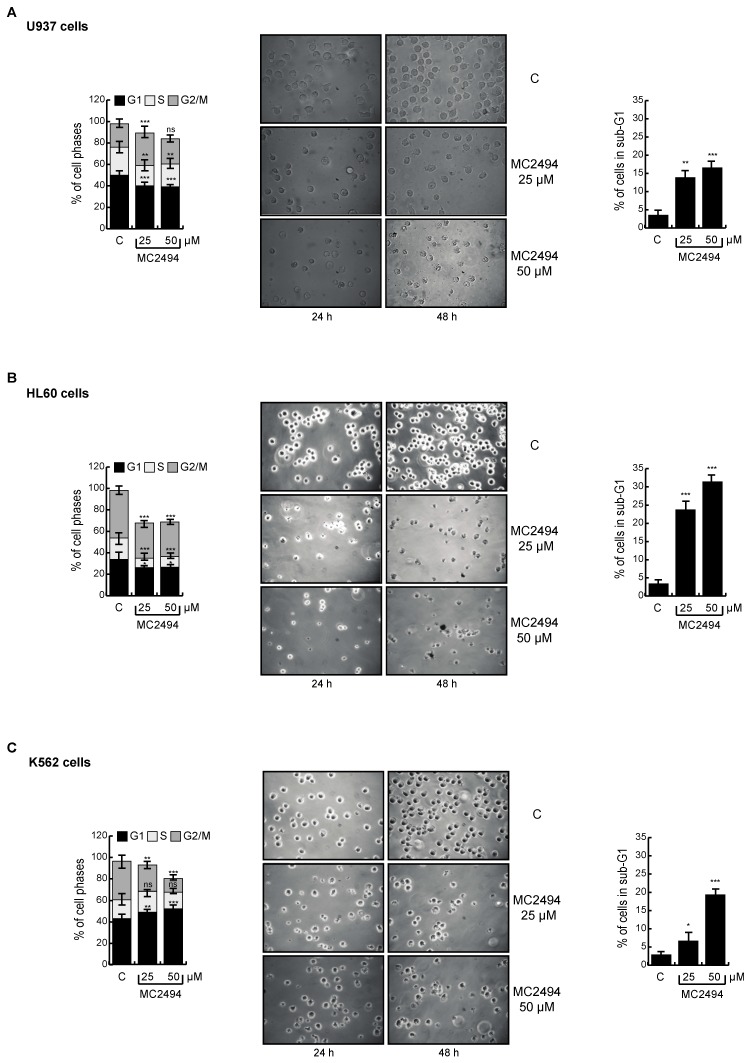
MC2494 affects proliferation in leukemia cancer cell lines. U937, HL60, and K562 cells were treated with MC2494 for 24 h and 48 h at two different concentrations (25 μM and 50 μM). Cell cycle analysis (left panels), morphological analysis performed with bright-field light microscopy (40×) (middle panels), sub-G1 analysis (right panels). (**A**) Experiments performed with U937 cells. (**B**) Experiments performed with HL60 cells. (**C**) Experiments performed with K562 cells. Graphs show the mean of at least two independent experiments with error bars indicating standard deviation. Values are mean ± SD of biological triplicates. *** *p*-value ≤ 0.001, ** *p*-value ≤ 0.01, * *p*-value ≤ 0.05, ns *p*-value > 0.05 vs. control cells.

Leukemia cell lines were induced only for 24 h with MC2494 and cell cycle was analyzed. In U937 cells, MC2494 determined a robust reduction in G1 phase detectable already at 25 μM concentration after 24 h of treatment (39.6%), and slight changes in S and G2/M phases (S, 20.6%; G2/M, 29.2%) (Figure 4A left panel). While MC2494 did not alter cell cycle progression in HL60 cells, in K562 cells we observed an increase in G1 phase already at 25 μM (49.4%), and a reduction in G2/M phase (24.6%). An even greater G2/M phase reduction (12.7%) was observed with the higher concentration of 50 μM (Figure 4B,C left panel). Analysis of the sub-G1 population revealed that U937 and HL60 cells were sensitive to MC2494 treatment already at 25 μM concentration (12.9% and 22.9%, respectively) (Figure 4A,B right panel). In contrast, in K562 cells MC2494 treatment induced an increase in sub-G1 phase (19.4%) only at 50 μM concentration (Figure 4C right panel).

In MCF7 cells, treatment with MC2494 (25 μM) determined an appreciable G1 phase arrest (60.9%) compared to control cells (51.8%) after 24 h (Supplementary Figure S6A left panel).

Further investigation of MDA-MB-231, MDA 453, and T47D cell lines revealed a reduction in G1 phase and a slight increase in G2/M phase after MC2494 treatment (50 μM). In MDA-MB-231 cells, the percentage of cells in G1 phase decreased (47.3%) after 24 h of treatment (Supplementary Figure S6B left panel). A slight effect on G1 phase was observed in T47D cells after 48 h of treatment with MC2494 at both concentrations (25 μM, 47.6%; 50 μM, 45.9%) (Supplementary Figure S7B left panel). Unlike the other cell lines, MDA 453 cells showed a decrease in cell population in G1 phase (48.9%) after 24 h of MC2494 treatment already at the lower concentration of 25 μM (Supplementary Figure S7A left panel). As regards the sub-G1 population, treatment with MC2494 at both concentrations (25 μM and 50 μM) induced cell death in T47D cells (8.8% and 8.9%, respectively), and at a final concentration of 50 μM induced an effect in MCF7 cells (10%) (Supplementary Figure S6A right panel and Supplementary Figure S7B right panel). A robust proliferation arrest and cell death were also detected by morphological analysis performed in all cancer cell lines investigated. Results obtained using bright field light microscopy (20X, breast cancer cells; 40X, leukemia cells) were consistent with the data presented, corroborating the previously reported finding that MC2494 reduces proliferation rate and viability in cancer cell lines (Figure 4 and Supplementary Figures S6 and S7). Cell death analyses highlighted that breast cancer cell lines were more resistant to MC2494 induction than leukemia cells (Figure 4 and Supplementary Figures S4 and S6). To confirm sub-G1 data, we also investigated PI intercalation in all cell lines after MC2494 treatment. After PI staining, a strong decrease in living cells was detected already after 24 h of treatment at 50 μM in MCF7 (15%), MDA-MB-231 (10%), MDA 453 (30%), T47D (20%), U937 (32%), HL60 (25%), and K562 (16%) cells (Supplementary Figure S8A,B).

### 2.5. MC2494 Regulates Cancer Cell Cycle Progression at Molecular Level

**Figure 5:**
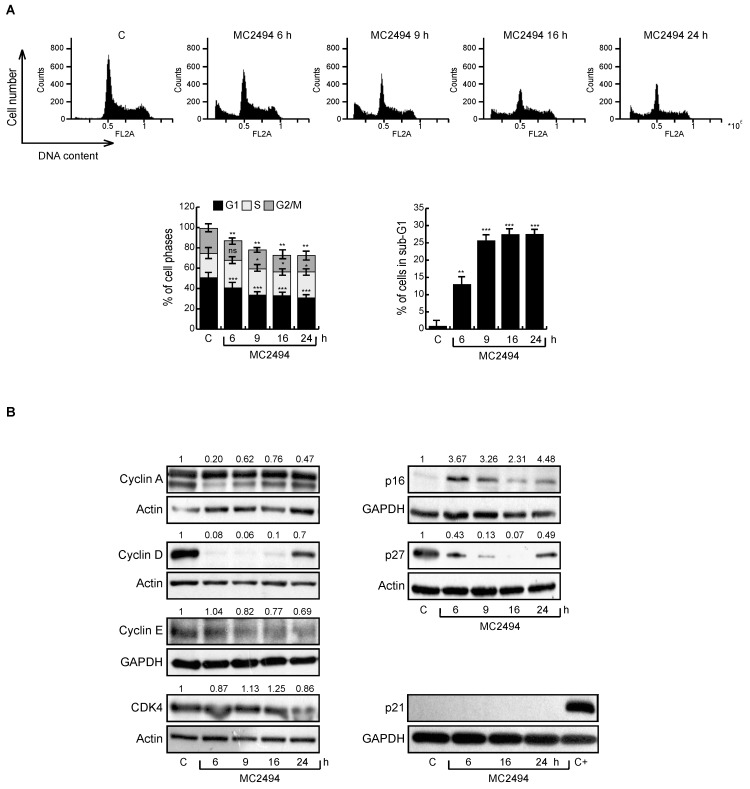
Effect of MC2494 on cell cycle regulation. U937 cells were treated with MC2494 for the indicated times at 50 μM. (**A**) Cell cycle distribution (top panel), cell cycle analysis (bottom left panel), sub-G1 analysis (bottom right panel). (**B**) Western blot analysis of indicated cell cycle regulatory proteins. Actin and GAPDH were used as controls for equal loading. Graphs show the mean of at least two independent experiments with error bars indicating standard deviation. Numbers on Western blot indicate the result of densitometry analysis, performed using the Image J Gel Analysis tool. Values are mean ± SD of biological triplicates. *** *p*-value ≤ 0.001, ** *p*-value ≤ 0.01, * *p*-value ≤ 0.05, ns *p*-value > 0.05 vs. control cells.

To further investigate the effect of MC2494 on cell cycle progression, U937 cells were induced with MC2494 at 25 μM at different treatment times (6–24 h). As expected, MC2494 reduced U937 cell viability, inducing an increase in sub-G1 phase in a time-dependent manner. In addition, the percentage of cells in G1 and G2/M phases decreased already after 6 h of treatment from 49% to 38.8% (G1) and from 22.4% to 17.9% (G2/M). This decrease was more appreciable at longer treatment times (Figure 5A). The action of MC2494 on cell cycle progression at molecular level was investigated by performing Western blot experiments on a panel of cell cycle-related proteins. Treatment with MC2494 led to a slight decrease in cyclin A and cyclin E, mostly at early treatment times (6 h and 9 h), and a significant reduction in cyclin D, indicating that the compound is able to block cell cycle progression (Figure 5B). The expression of CDK4 was unaltered, while p27 expression decreased after treatment and p21 protein expression was not detectable. In contrast, protein levels of p16 increased, indicating that MC2494 interacts with p16 causing a block at early stages of the cell cycle (Figure 5B).

Cell cycle analysis was also performed in MCF7 cells, revealing an opposite effect to that observed in U937 cells, with a higher percentage of cells blocked in G1 phase following MC2494 treatment. This increase was observed already after 6 h of treatment, with the greatest effect after 24 h (from 48.6% to 56.5%). MC2494 induced a slight decrease in cyclin D and p21, accompanied by an increase in expression of cyclin E after 9 h and 16 h of treatment (Supplementary Figure S9).

**Figure 6:**
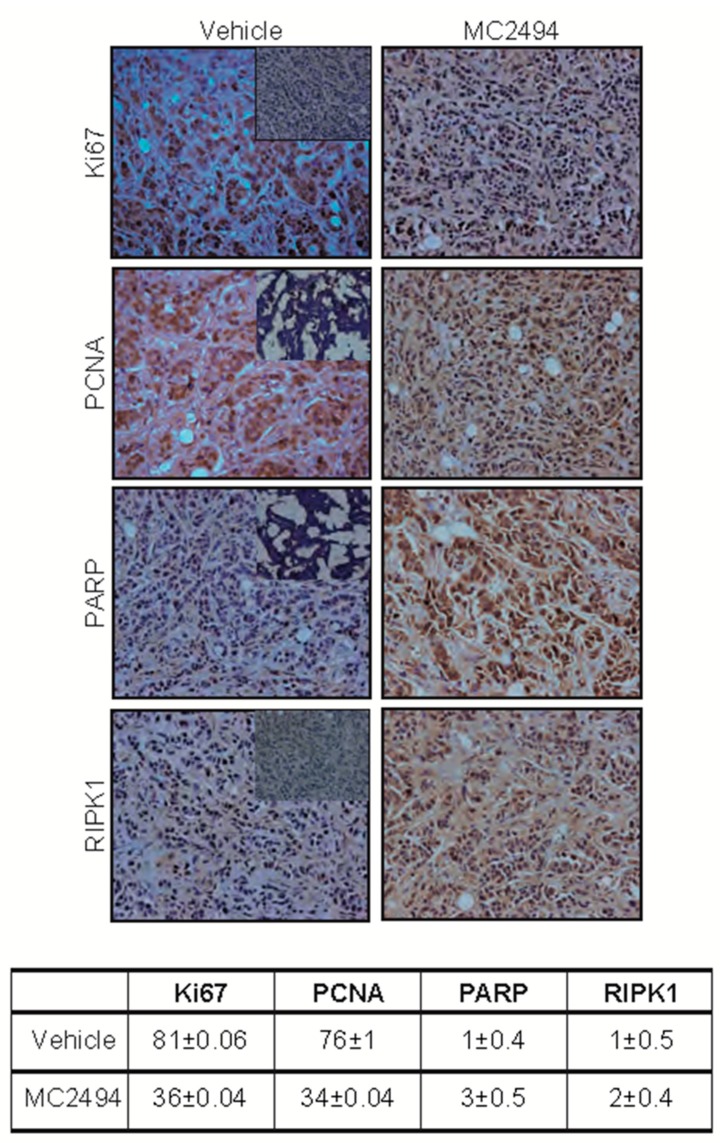
MC2494 displays broad in vivo anticancer action. Immunohistochemistry analysis for PCNA, Ki67, RIPK1, and PARP levels in tumor. Score and statistical analysis are reported in the table in the bottom panel. Negative controls are included as inserts in each panel for all antibodies. (Objective lenses 10×).

Following MC2494 treatment, a block of proliferation Proliferating cell nuclear antigen [PCNA] and Ki67 and an induction of cell death (PARP and RIPK1) were observed after immunohistochemistry analysis performed on murine breast tumor specimens derived from allograft BALB/c mice (Figure 6), supporting the data obtained in cell lines and extending previous results [28].

### 2.6. MC2494 Impacts on Cancer Cell Migration

**Figure 7:**
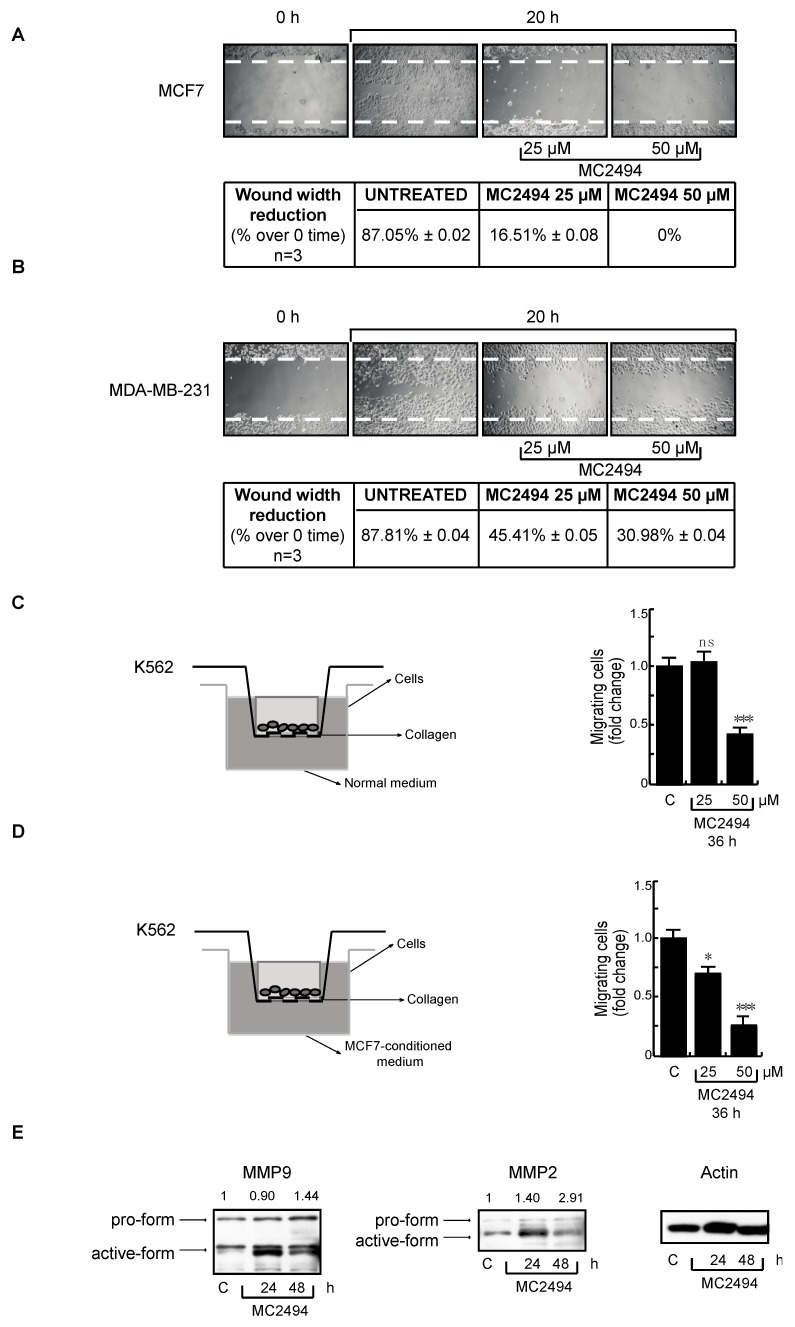
MC2494 affects cell migration. (**A**) Wound-healing assay performed in MCF7 cells at indicated times and concentrations. (**B**) Wound-healing assay performed in MDA-MB-231 cells at indicated times and concentrations. (**C**) Migration analysis using Boyden’s chamber system performed in K562 cells. (**D**) Migration analysis using Boyden’s chamber system performed in K562 cells after treatment with conditioned medium. (**E**) Western blot analysis of MMP2 and MMP9. Actin was used as control for equal loading. Values are mean ± SD of biological triplicates. *** *p*-value ≤ 0.001, * *p*-value ≤ 0.05, ns *p*-value > 0.05 vs. control cells.

In previous works, we reported that SIRT1 activation by 1,4-dihydropyridine compounds ameliorated wound healing by increasing cell migration, while the SIRT inhibitor sirtinol had an opposite effect [29,30,31]. To investigate whether MC2494 affects cell migration, we treated MCF7 and MDA-MB-231 cells at two different concentrations (25 μM and 50 μM) for 20 h and performed wound-healing experiments. Representative images from one experiment were captured by contrast-phase microscopy (Figure 7A,B). The wound width from different experiments was analyzed as a reduction in wound area compared to 0 time. Our results show that a significant number of cells migrated to the wound area in cycling cells and that MC2494 already inhibited cell migration at a concentration of 25 μM in both MCF7 and MDA-MB-231 cells. This MC2494-mediated effect was comparable to inhibition due to serum deprivation.

To further assess the effect of MC2494 on cell migration, K562 cells were treated with MC2494 at 25 μM and 50 μM for 36 h and analyzed with Boyden’s chamber system (Figure 7C,D). MC2494 treatment at 50 µM inhibited by 2-fold the number of migrating K562 cells. U937 cells were not used for these experiments due to their high sensitivity to MC2494 treatment in terms of cell death. To confirm these data, conditioned medium from MCF7 cells was added in the lower chamber of the Transwell system. Several studies showed that the addition of conditioned medium promotes cell migration. In these experimental conditions, we observed a drastic reduction in the number of migrating K562 cells after 36 h of MC2494 treatment (Figure 7C,D). To confirm this finding, Western blot analysis performed for two metalloproteinases (MMP2 and MMP9) involved in cell migration revealed that together with the block of proliferation an accumulation of the metalloproteinase inactive forms (pro-MMP2 and pro-MMP9) occurred (Figure 7E).

These findings indicate that SIRT inhibition mediated by MC2494 has a functional role in cancer cell migration.

## 3. Discussion

SIRT1, the most characterized SIRT, is involved in many vital biological processes and plays a critical role in cancer initiation, promotion, and progression. Based on the chemical scaffold of MC2494, we developed a small library (21 compounds) of related derivatives, which were then characterized at enzymatic and biological level. Firstly, by treating leukemia U937 cells with the analogs and comparing their biological activity to that induced by MC2494, we detected a decrease in the expression level of SIRT1 and SIRT2. This action did not correlate with the effect of the same compounds when tested in vitro for SIRT inhibition. These findings indicate that, depending on the experimental setting, the investigated compounds display different functions and that in vitro assays may not always recapitulate the action in cells, mimicking the mechanism of SIRT inhibition.

A preliminary SAR study underlined a switch of selectivity from pan-inhibition to SIRT2-inhibition, especially for compounds 17 and 18, suggesting that further optimization of the MC2494 chemical scaffold could be a start point for synthesis of new MC2494-related compounds. To further characterize the novel compounds, we evaluated the effects of MC2494 analogs on H3K9/14ac and acetyl tubulin, two of the main targets of SIRT1 and SIRT2 deacetylation, respectively. The data obtained supported the results of the enzymatic assay, suggesting that the downregulation of SIRT expression induced by some of the compounds is not directly dependent on their enzymatic inhibition. Moreover, the cyano moiety substitution with a carbethoxy group in portion C resulted in a marked inhibition of SIRT2, highlighting the significant change in the selectivity profile of the pan-inhibitor MC2494 prototype.

Data obtained via analysis of cell proliferation and apoptosis induced by MC2494 derivatives corroborated the activity of MC2494, warranting further studies.

To better investigate the antiproliferative mechanism(s) of MC2494 in different systems, we studied its action in a broad panel of cancer cell lines, particularly comparing hematological (leukemia) and solid (breast) cancers. Given conflicting reports on the association between SIRT1 and p53 [32,33], we chose cell systems with different p53 expression and mutational status. Our study revealed that among the three leukemia cell lines used, U937 cells were more sensitive than HL60 and K562 cells to MC2494-induced cell death, already at the lower concentration of 25 μM. In contrast, breast cancer cell lines were generally more resistant to MC2494 treatment, which resulted in a low percentage of sub-G1 populations. Interestingly, MCF7 cells were more sensitive than all other breast cancer cell lines, suggesting the potential activation of different biological pathways via SIRT inhibition [34].

Of note, results obtained in different cell lines from sub-G1 evaluation and PI staining following MC2494 treatment showed different responses, particularly in breast cancer cells (which were positive to PI staining but displayed a low sub-G1 fraction). This observation suggests that the modulation of early as well as late apoptotic pathways varies in a context-dependent manner. Further studies may clarify whether in breast cancer cells the SIRT1 inhibitory pathway contributes to this delayed apoptosis and the role of context specificities.

We speculated that SIRT inhibition may act by interfering with cell cycle phases, given that SIRTs impact on cell cycle progression. Cell cycle and sub-G1 phases were affected by SIRT inhibition induced by MC2494, with leukemia U937 and breast-cancer MCF7 cells exhibiting a different regulation. The effect on MCF7 cells was in line with previously reported findings for another SIRTi [35], determining an increase in G1 phase via decreased expression of cyclin D1 (6 h) and p21 (6 h, with a peak at 16 h).

In contrast, p21 expression was not detected in U937 cells even after MC2494 treatment. The inhibition of SIRT1 determined a significant decrease in cyclin D1 and to some extent in cyclin E, with an increase in p16, suggesting that in U937 cells the block of cell cycle occurs via p16. These data strengthen the context-specific action of SIRT inhibition even at the level of cell cycle regulation, suggesting that the antiproliferative effects of MC2494 might be obtained by activating different regulatory pathways of cell cycle progression. Given the functional role of SIRTS in cell migration and motility [36], SIRT inhibition induces a decrease in cell migration and motility. It is tempting to speculate that cells which seem to be more resistant to the induction of cell death upon MC2494 treatment may be particularly prone to activating programs blocking migration/motility. Further investigation will be needed to determine whether this is indeed the case and what the underlying molecular basis might be. The observed activity of MC2494 via regulation of cell cycle, apoptosis, and inhibition of cell migration supports the potential role of SIRTs as a target in tumorigenesis and makes SIRT-targeting molecules good candidates for novel pharmacological approaches in personalized medicine.

## 4. Materials and Methods

### 4.1. Ligands

MC2494 was prepared as reported previously [28]. The detailed preparation and chemical-physical characterization of MC2494 analogs 1–21 will be reported elsewhere in another publication. MC2494 (and derivatives) were dissolved in dimethyl sulfoxide (DMSO) and used at 5 × 10^−5^ M. EX527 (Alexis) was dissolved in DMSO (Sigma-Aldrich) and used at 5 × 10^−6^ M. AGK was dissolved in DMSO and used at 5 × 10^−5^ M.

### 4.2. Morphological Analysis

Morphological analysis for MCF7, MDA-MB-231, MDA 453, and T47D cancer cell lines was performed using bright field light microscopy (20×). For U937, HL60, and K562 cancer cell lines, the analysis was performed using bright-field light microscopy (40×).

### 4.3. Antibodies

Cyclin A, cyclin D, cyclin E, CDK4, p27, p21, and tubulin were from Santa Cruz. MMP2, MMP9, p16, and histone H4 were from Abcam, GAPDH from Cell Signaling, and histone H3 K9/14ac from Diagenode.

### 4.4. Cell Lines

U937, HL-60, and K562, cells were purchased from DSMZ. MDA-MB-231, MDA 453, MCF-7, and T47D cells were from Cell Bank Interlab Cell Line Collection. HEK293T and HaCaT cells were from Thermo Fisher Scientific. All cell lines and primary cells were grown following standard protocols at 37 °C with 5% CO_2_. Human leukemia U937, HL-60, and K562 cells were propagated in Roswell Park Memorial Institute (RPMI) 1640 medium, with 10% fetal bovine serum (FBS; Gibco), 2 mM L-glutamine (Euroclone), and antibiotics (100 U/mL penicillin, 100 μg/mL streptomycin, and 250 ng/mL amphotericin-B; Euroclone). Human breast cancer MCF7 and MDA-MB-231 cells were propagated in Dulbecco’s Modified Eagle Medium (DMEM) medium, 2 mM L-glutamine (Euroclone), 10% FBS, and antibiotics. T47D cells were propagated in RPMI with 10% FBS, 2 mM L-glutamine (Euroclone), and antibiotics. MDA 453 cells were propagated in DMEM-F12 medium, 2 mM L-glutamine (Euroclone), 10% FBS, and antibiotics. Human epithelial kidney HEK293FT cells were propagated in RPMI with 10% FBS and antibiotics, 0.1 mM MEM Non-Essential Amino Acids, 6 mM L-glutamine (Euroclone), and 1 mM MEM sodium pyruvate. Human keratinocyte HaCaT cells were propagated in DMEM medium, 2 mM L-glutamine (Euroclone), 10% FBS, and antibiotics. Mycoplasma contamination was regularly examined using EZ-PCR Mycoplasma Test Kit (Biological Industries). All cell lines were tested and authenticated. Cells were used for experiments between passages 10 to 20 and then discarded.

### 4.5. SIRT1 Purification

SIRT1-GST enzyme (glutathione S-transferase) was purified by *E. coli* BL21 bacteria after transfection with pGEX-SIRT1 (Addgene) plasmid. One selected bacterial colony was grown in LB broth medium (Lennox) supplemented with antibiotics (100 μg/mL ampicillin) in a shaking incubator overnight. When optical density was in a range between 0.6 and 0.8, protein expression was induced by isopropyl-β-D-1-thiogalactopyranoside (AppliChem) at 200 μM concentration for 5 h. The bacteria were centrifuged at 1381 rcf (Beckman centrifuge) and the pellet was then lysed by sonication (Sonic Diagenode). Lysis buffer was composed of phosphate buffered saline (PBS), 1 mM 1,4-dithiothreitol (DTT; Applichem), 0.5 mM phenylmethylsulfonyl fluoride (AppliChem), and 1 tablet of mini protease inhibitor cocktail (PIC; Roche) for each 10 mL. The bacteria were sonicated for 10 cycles of 45 sec at 14 000 MHz with intervals of 30 sec between each sonication. Then, Triton X-100 0.1% (Acros) was added followed by incubation for 15 min in ice. The sonicate was then centrifuged at 17761rcf (Centrifuge 5430 R; Eppendorf) for 30 min and filtered with a filter of 0.45 μm pore size. The bacterial lysate was purified using GSTrap 4B columns (GE Healthcare Life Sciences). The columns were equilibrated with 20 mL lysis buffer. Next, the lysate was loaded onto columns and subsequently they were washed with the lysis buffer. The elution was carried out with 20 mL elution buffer composed of 50 mM Tris- HCl pH 8.0, 1 mM DTT, 20 mM L-glutathione reduced (AppliChem), and ddH_2_O. SIRT1-GST protein was detected using colorimetric methods (Bradford protein assay; Bio-Rad). Twenty-five μL of each eluate collected from purification were diluted in Laemmli sample buffer 6X (0.217 M Tris-HCl pH 8.0, 52.17% Sodium dodecyl sulfate (SDS), 17.4% glycerol, 0.026% bromophenol blue, 8.7% β-mercaptoethanol), and then boiled for 5 min. Twelve eluates were run and separated on 10% acrylamide gel. After the run, the gels were colored with Coomassie Blue and bleached with destaining solution (35% methanol, 15% acetic acid in distilled H_2_O). Dialysis was performed using a buffer composed of 50 mM Tris-HCl pH 8.0, 100 mM NaCl (Sigma-Aldrich), 1 mM DTT, 1 tablet of PIC (for each 10 mL), and ddH_2_O overnight at 4 °C. The following day, another dialysis was performed for 2 h. Finally, the samples were cryopreserved in 20% glycerol (Sigma-Aldrich).

### 4.6. SIRT Assays

The SIRT1 assay is a fluorimetric assay that uses a substrate (Fluor de Lys-SIRT1) recognized and deacetylated by SIRT1 in the presence of NAD^+^, with fluorescence emission. The Fluor de Lys-SIRT1 substrate is a peptide built on the amino acid sequence of human p53, which consists of amino acids 379–382 (Arg-His-Lys-Lys[Ac]). The assay was performed in a 96-well microtiter plate reader with fluorescent readout (Corning 96 flat bottom black polystyrene). The final reaction volume was 25 μL. The reaction buffer was composed of PBS and 1 mM DTT. All compounds were dissolved in DMSO and tested at a concentration of 50 μM. SIRT1-purified enzyme (5 μL) at a dilution of 1 mg/mL was incubated for 15 min at 37 °C with 5 μL intermediate dilution (50 μM) of compounds or 5 μL reaction buffer with 0.6% DMSO for positive control. A mix composed of 5 μL nicotinamidase (NMase-purified enzyme), 5 μL β-NAD intermediate dilution (1 mM) and 5 μL acetylated peptide p53K382 intermediate dilution (250 μM; synthesized by INBIOS) was then added and the whole mix was incubated for 40 min at 37 °C. Subsequently, developer buffer (70% PBS, 30% ethanol, 10 mM DTT, and 10 mM o-phthalaldehyde [OPT; Acros]) was added, followed by re-incubation for 30 min at room temperature. Fluorescent signal detection was performed with an Infinite M200 Tecan microplate reader at 420/460 nm. This assay correlates SIRT1 deacetylase activity with production (and quantification) of ammonia by coupling two reactions catalyzed by SIRT1 and NMase. In the first reaction, SIRT1 removes the acetyl group from the lysine in position 382 of the p53 peptide (amino acids 374–389) via reaction with its cofactor NAD^+^, which is cleaved forming O-acetyl-ADP-ribose and NAM. In the second reaction, the NMase enzyme converts NAM into nicotinic acid and free ammonia. Finally, ammonia is detected as a fluorescent adduct at 420/460 nm, in presence of OPT present in the stop solution. The fluorescent signal is generated in proportion to the amount of deacetylation. SIRT2 assay was performed by Reaction Biology Corporation.

### 4.7. Cell Cycle and Cell Death Analysis

For cell cycle analyses, cells were plated (2 × 10^5^ cells/mL) and after stimulation (performed as described in the main text) were harvested with PBS, centrifuged at 1200 rpm for 5 min, and resuspended in 500 μL of a hypotonic solution containing 1X PBS, 0.1% sodium citrate, 0.1% NP-40, RNAase A, and 50 mg/mL PI. Cell death was studied by evaluating hypodiploid sub-G1 peak on fixed cells and PI incorporation on live cells to assess DNA fragmentation (late apoptotic event) and dead-cell membrane permeabilization (early apoptotic event), respectively. For sub-G1 evaluation, samples were prepared as described above. For PI evaluation, cells were plated (2 × 10^5^ cells/mL) and stimulated for 24 h. After stimulation, cells were harvested with PBS, centrifuged at 1200 rpm for 5 min, and resuspended in 500 μL 1X PBS and 0.2 mg/mL PI. The results were acquired on a BD Accuri TM C6 flow cytometer system (BD Biosciences). Each experiment was performed in biological triplicates and values expressed as mean ± standard deviation.

### 4.8. Annexin V Staining

Annexin V evaluation, was performed as suggested by the supplier (Dojindo). Briefly, suspension cells were suspended in Annexin V binding solution at the final concentration of 1 × 10^6^ cells/mL. 100 μL of this suspension was transferred into a new tube, and Annexin V Fluorescein isothiocyanate (FITC) conjugated and PI were added. Reaction was carried out for 15 min at room temperature. The results were acquired on a BD Accuri TM C6 flow cytometer system (BD Biosciences). Graphs show the experimental results of biological triplicates.

### 4.9. Protein Extraction

After PBS wash, cell pellets were suspended in lysis buffer (50 mM Tris-HCl pH 7.4, 150 mM NaCl, 1% NP-40, 10 mM NaF, 1 mM phenylmethylsulfonyl fluoride (PMSF), and protease inhibitor (PIC). The lysis reaction was carried out for 15 min at 4 °C. Finally, the samples were centrifuged at 13,000 rpm for 30 min at 4 °C and protein concentration quantified by Bradford assay (Bio-Rad).

### 4.10. Western Blot

Fifty μg of proteins was loaded onto 10–15% polyacrylamide gels. Between 5 and 10 μg of histone extract was loaded onto 15% polyacrylamide gel. The nitrocellulose filters were stained with Ponceau Red (Sigma-Aldrich) as additional control for equal loading. Whole cell extracts were transferred to a nitrocellulose membrane using a transfer apparatus according to the manufacturer’s protocols (Bio-Rad). After incubation with 5% non-fat milk in TBST (10 mM Tris pH 8.0, 150 mM NaCl, 0.5% TWEEN 20) for 60 min, the membrane was washed once with TBST and incubated with antibodies. Detection was performed with an Enhanced chemiluminescence (ECL) system (Amersham Biosciences) according to the manufacturer’s protocol.

### 4.11. In Vivo Experiments

For in vivo studies, 2.5 × 10^5^ 4T1-Luc cells suspended in 100 μL of PBS, were injected in the mammary fat pad of six-week-old female athymic BALB/c mice.

Two different experimental groups were generated (control and MC2494-treated), each consisting of seven mice. Mice were treated through intraperitoneal injection 6 days/week, using 50 mg/kg/day of MC2494 concentration or DMSO alone for control group. Tumor growth evaluation was performed after 7 days (T7) from the start of treatment and immunochemistry experiments were performed following the method described above.

### 4.12. Immunohistochemistry

The excised biopsies were fixed in 10% buffered formalin and paraffin embedded. Sections of 5 μ were incubated in a microwave oven for 15 min in 10 mmol/L pH 6.0 buffered citrate followed by the immunohistochemical procedure for Ki67 (Santa Cruz), PCNA (Abcam), PARP (Cell Signaling) and RIP1 (BD Biosciences) diluted 1:100. For Ki67 and PCNA detection, the incubation of primary antibody was performed over night at 4 °C. The conventional avidin–biotin complex procedure was applied according to the manufacturer’s protocol (Dako) and then incubated with a secondary antibody. Positive staining was revealed by DAB chromogen, according to the supplier’s instructions.

### 4.13. Wound-Scratch Assay

For wound-scratch assay, 1.5 × 10^5^ cells were seeded in a 24-well plate. Cells were wounded using 10 µL sterile pipette tips. Cells were washed with PBS and then untreated or treated for the indicated times in the absence or presence of different concentrations of MC2494, as indicated in Supplementary Figure S8. Cytosine arabinoside (Sigma-Aldrich) at 50 µM (final concentration) was included in the cell medium to avoid cell proliferation. Different fields were analyzed using a DM IRB inverted microscope (Leica) equipped with an N-Plan 10x objective (Leica) and Application Suite Software (Leica), as reported [37]. Contrast-phase images, representative of at least three different experiments, were captured using a DC200 camera (Leica). The wound gap was calculated using Image J software and expressed as a percentage of the decrease in wound area, as described [38].

### 4.14. Boyden’s Chamber Assay

Boyden’s chamber assay was performed using collagen-I from rat-tail (BD Biosciences)-coated Transwells with 8 μm polycarbonate membrane (Corning) [38]. Cycling cells were plated in the upper chamber at 5 × 10^4^ per well in 150 µL complete medium.

When indicated, MCF-7 conditioned medium was added to the lower chambers. Cells were allowed to migrate for 36 h in a humidified incubator at 37 °C with 5% CO_2_ in the absence or presence of the compound added to the upper and lower chambers at the indicated concentrations.

After 36 h, non-migrating cells from the membrane upper surface were removed using a sterile cotton swab. The polycarbonate membranes were fixed for 20 min in 4% paraformaldehyde, stained with Hoechst, removed with forceps from the companion chamber and mounted. Migrating cells from at least 30 fields/each membrane were counted as described [38], using a DMBL fluorescent microscope (Leica), equipped with an HCPL Fluotar 20× objective. Data are representative of three different experiments.

### 4.15. Statistical Analysis

Graphs shown in the figures represent the mean of three independent experiments with an error bar indicating standard deviation. Differences between treated cells versus control cells were analyzed using GraphPad Prism 6.0 software (GraphPad Software, Inc., San Diego, CA, USA). Statistical comparison was performed by applying one-way analysis of variance (ANOVA) and Dunnett’s multiple-comparison test. Differences between groups were considered to be significant at a *p*-value of < 0.05.

## 5. Conclusions

Our investigations into MC2494-related compounds indicated that the chemical substitutions applied to MC2494 scaffold did not confer higher efficacy in terms of biological activity and SIRT1 inhibition compared to the prototype. However, the carbethoxy-containing derivatives showed higher specificity toward SIRT2. The strongest enzymatic activity was observed with the carbethoxy derivative of MC2494 and its 2-methyl analog (compound 17 and 18, respectively). These findings suggest that the applied chemical modifications improved the enzymatic selectivity of these compounds. In addition, the observed activity of MC2494 via regulation of cell cycle, apoptosis, and inhibition of cell migration supports the potential role of SIRTs as a target in tumorigenesis and makes SIRT-targeting molecules good candidates for novel pharmacological approaches in personalized medicine.

## Figures and Tables

**Table 1:** Structure–activity relationship (SAR) studies of MC2494. Structure of MC2494 derivatives 1–21.

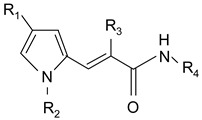				
**Compd**	**R_1_**	**R_2_**	**R_3_**	**R_4_**
**MC2494**	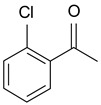	H	CN	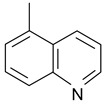
**1**	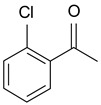	CH_3_	CN	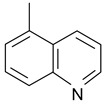
**2**	H	H	CN	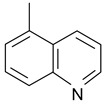
**3**	H	CH_3_	CN	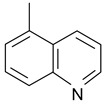
**4**	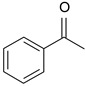	H	CN	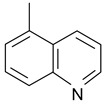
**5**	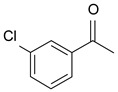	H	CN	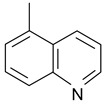
**6**	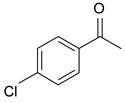	H	CN	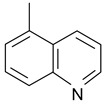
**7**	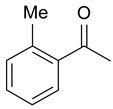	H	CN	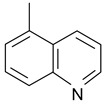
**8**	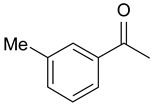	H	CN	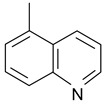
**9**	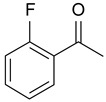	H	CN	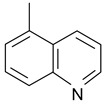
**10**	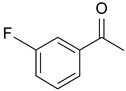	H	CN	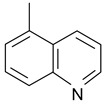
**11**	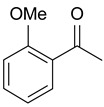	H	CN	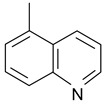
**12**	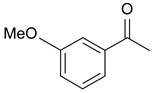	H	CN	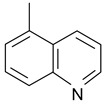
**13**	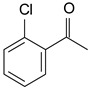	H	CN	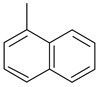
**14**	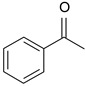	H	CN	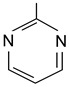
**15**	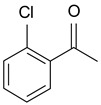	H	CN	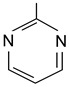
**16**	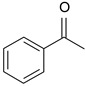	H	COOEt	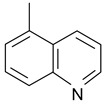
**17**	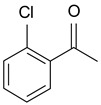	H	COOEt	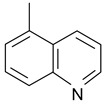
**18**	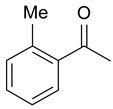	H	COOEt	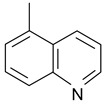
**19**	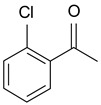	H	COOEt	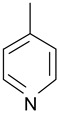
**20**	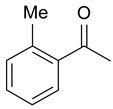	H	COOEt	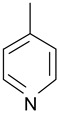
**21**	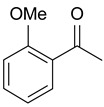	H	COOEt	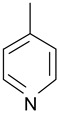

## Supplementary Materials

Supplementary materials can be found at https://www.mdpi.com/1422-0067/20/22/5654/s1.
