# Supplementary material for: Enzymatic and Biological Characterization of Novel Sirtuin Modulators against Cancer
Source: Int J Mol Sci. 2019 Nov 12;20(22):5654. doi: 10.3390/ijms20225654 (PMC6888689; doi:10.3390/ijms20225654)
Supplement: Supplementary file 1 [file ijms-20-05654-s001.pdf]

Supplementary Materials

Enzymatic and biological characterization of novel sirtuin modulators against cancer

Vincenzo Carafa[1], Angelita Poziello[1], Laura Della Torre[1], Pia Giovannelli[1], Marzia Di Donato[1], Elham Safadeh[1], Zhijun Yu[1], Alfonso Baldi[2], Gabriella Castoria[1], Daniela Tomaselli[3], Antonello Mai[3*], Dante Rotili[3*], Angela Nebbioso[1*] and Lucia Altucci[1*]

[1] Dipartimento di Medicina di Precisione, Università degli Studi della Campania "Luigi Vanvitelli", 80138 Napoli, IT; vincenzo.carafa@unicampania.it; angelita.poziello@unicampania.it; laura.dellatorre@unicampania.it; pia.giovannelli@unicampania.it; marzia.didonato@unicampania.it; elham.safadeh@unicampania.it; zhijun.yu@unicampania.it; gabriella.castoria@unicampania.it; angela.nebbioso@unicampania.it; lucia.altucci@unicampania.it

[2] Dipartimento di Scienze e Tecnologie Ambientali Biologiche e Farmaceutiche Università degli Studi della Campania "Luigi Vanvitelli", 81100 Caserta, IT; alfonso.baldi@unicampania.it

[3] Dipartimento di Chimica e Tecnologie del Farmaco "Sapienza" Università di Roma, 00185 Roma, IT; daniela.tomaselli@uniroma1.it. antonello.mai@uniroma1.it; dante.rotili@uniroma1.it

**Supplementary Table S1.** Preparation Yield, Purity, MS and NMR data of compounds 1-21.					
**Compd**	**Structure**	**Yield** (%)	**Purity**[a] (%)	**MS**[b] [M + H]$^+$	**^{1}H-NMR**[c]
**1**	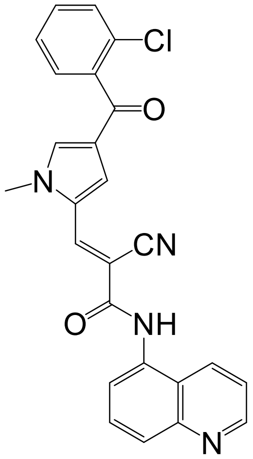	93	>95	441	^{1}H-NMR (DMSO) δ 3.85 (s, 3H, C*H*$_3$), 7.44-7.79 (m, 9H, pyrrole, benzene and quinoline protons), 7.94 (d, 1H, quinoline proton), 8.22 (s, 1H, C*H*=C(CN)CONH), 8.36 (d, 1H, quinoline proton), 8.91 (d, 1H, quinoline proton), 10.51 (s, 1H, CON*H*).
**2**	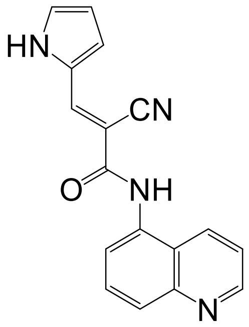	91	>95	289	^{1}H-NMR (DMSO) δ 6.49 (t, 1H, pyrrole proton), 7.39-7.43 (m, 2H, pyrrole protons), 7.56-7.59 (q, 1H, quinoline proton), 7.66-7.68 (d, 1H, quinoline proton), 7.76-7.80 (t, 1H, quinoline proton), 7.95-7.97 (d, 1H, quinoline proton), 8.22 (s, 1H, C*H*=C(CN)CONH), 8.34 (d, 1H, quinoline proton), 8.93-8.94 (d, 1H, quinoline proton), 10.29 (br s, 1H, CON*H*), 11.99 (br s, 1H, pyrrole N*H*).
**3**	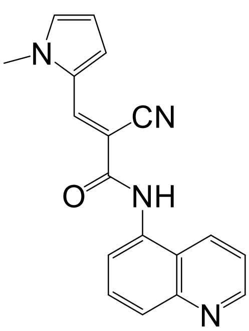	88	>95	303	^{1}H-NMR (DMSO) δ 3.80 (s, 3H, C*H*$_3$), 6.38 (t, 1H, pyrrole proton), 7.35 (m, 1H, pyrrole proton), 7.47 (d, 1H, pyrrole proton), 7.53-7.56 (q, 1H, quinoline proton), 7.62-7.64 (d, 1H, quinoline proton), 7.74-7.78 (t, 1H, quinoline proton), 7.91-7.93 (d, [illegible]

					(d, 1H, quinoline proton), 8.90-8.91 (d, 1H, quinoline proton), 10.34 (br s, 1H, CON*H*).
**4**	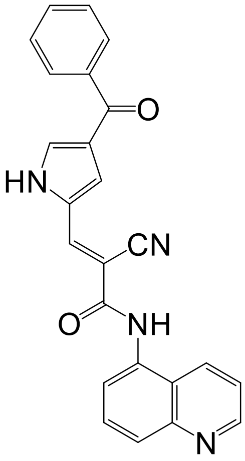	84	>95	393	^{1}H-NMR (DMSO) δ 7.56-7.69 (m, 5H, pyrrole, benzene and quinoline protons), 7.78-7.99 (m, 6H, pyrrole, benzene and quinoline protons), 8.29 (s, 1H, C*H*=C(CN)CONH), 8.37 (d, 1H, quinoline proton), 8.94-8.95 (d, 1H, quinoline proton), 10.43-10.53 (br s, 1H, CON*H*), 12.56-12.66 (br s, 1H, pyrrole N*H*).
**5**	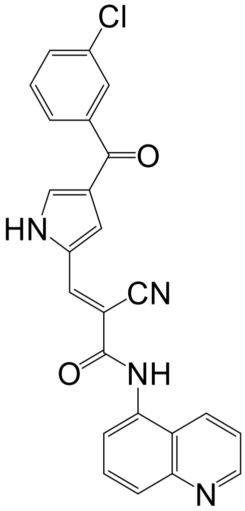	85	>95	427	^{1}H-NMR (DMSO) δ 7.58-7.80 (m, 8H, pyrrole, benzene and quinoline protons), 7.96-7.99 (m, 2H, benzene and quinoline protons), 8.28 (s, 1H, C*H*=C(CN)CONH), 8.36-8.39 (d, 1H, quinoline proton), 8.95 (br d, 1H, quinoline proton), 10.44-10.58 (br s, 1H, CON*H*), 12.59-12.74 (br s, 1H, pyrrole N*H*).
**6**	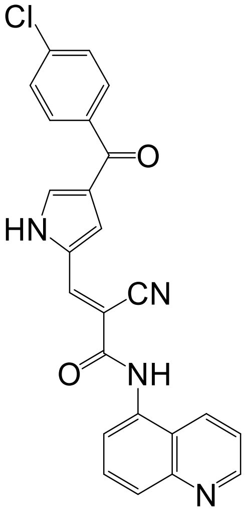	75	>95	427	^{1}H-NMR (DMSO) δ 7.57-7.87 (m, 8H, pyrrole, benzene and quinoline protons), 7.95-7.99 (m, 2H, benzene and quinoline protons), 8.28 (s, 1H, C*H*=C(CN)CONH), 8.37 (d, 1H, quinoline proton), 8.95 (d, 1H, quinoline proton), 10.49 (br s, 1H, CON*H*), 12.50-12.78 (br s, 1H, pyrrole N*H*).

**7**	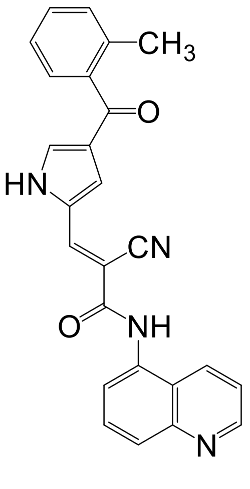	78	>95	407	^{1}H-NMR (DMSO) δ 2.31 (s, 3H, C*H*3), 7.31-7.46 (m, 4H, pyrrole and benzene protons), 7.57-7.69 (m, 4H, pyrrole, benzene and quinoline protons), 7.79 (t, 1H, quinoline proton), 7.96-7.98 (d, 1H, quinoline proton), 8.26 (s, 1H, C*H*=C(CN)CONH), 8.36 (d, 1H, quinoline proton), 8.94 (m, 1H, quinoline proton), 10.47 (s, 1H, CON*H*), 12.56 (s, 1H, pyrrole N*H*).
**8**	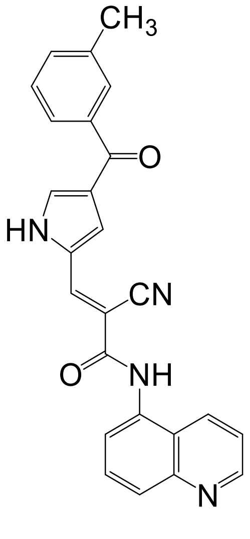	80	>95	407	^{1}H-NMR (DMSO) δ 2.42 (s, 3H, C*H3*), 7.45-7.47 (m, 2H, pyrrole and benzene protons), 7.57-7.69 (m, 4H, pyrrole, benzene and quinoline protons), 7.78-7.82 (m, 2H, benzene and quinoline protons), 7.93-7.99 (m, 2H, quinoline protons), 8.29 (s, 1H, C*H*=C(CN)CONH), 8.37 (d, 1H, quinoline proton), 8.95 (d, 1H, quinoline proton), 10.47 (br s, 1H, CON*H*), 12.52-12.65 (br s, 1H, pyrrole N*H*).
**9**	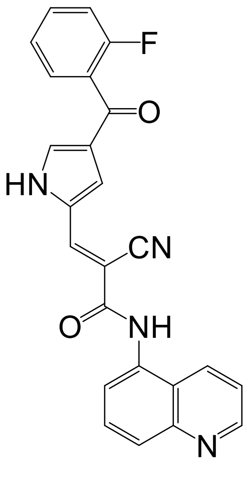	82	>95	411	^{1}H-NMR (DMSO) δ 7.35-7.41 (m, 2H, pyrrole and benzene protons), 7.57-7.68 (m, 4H, pyrrole, benzene and quinoline protons), 7.72-7.83 (m, 3H, benzene and quinoline protons), 7.96-7.98 (m, 1H, quinoline proton), 8.26 (s, 1H, C*H*=C(CN)CONH), 8.35-8.37 (d, 1H, quinoline proton), 8.94-8.95 (d,

					1H, quinoline proton), 10.46-10.56 (br s, 1H, CON*H*), 12.60-12.70 (br s, 1H, pyrrole N*H*).
**10**	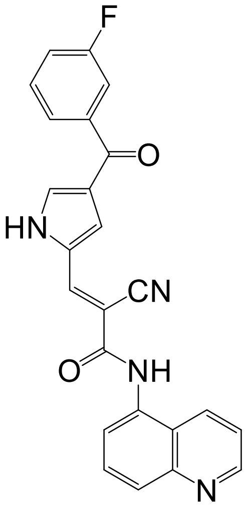	69	>95	411	^{1}H-NMR (DMSO) δ 7.50-7.68 (m, 6H, pyrrole, and benzene protons), 7.78-7.82 (m, 2H, quinoline protons), 7.97-7.99 (m, 2H, quinoline protons), 8.28 (s, 1H, C*H*=C(CN)CONH), 8.37 (d, 1H, quinoline proton), 8.95 (br d, 1H, quinoline proton), 10.44-10.54 (br s, 1H, CON*H*), 12.51-12.65 (br s, 1H, pyrrole N*H*).
**11**	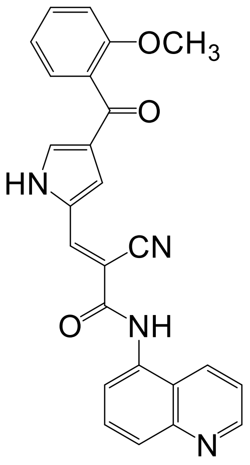	77	>95	423	^{1}H-NMR (DMSO) δ 3.77 (s, 3H, OCH_3), 7.07 (t, 1H, benzene proton), 7.19 (d, 1H, benzene proton), 7.34 (d, 1H pyrrole proton), 7.50-7.60 (m, 2H, quinoline protons), 7.65-7.69 (m, 3H, pyrrole, benzene and quinoline protons), 7.77-7.81 (t, 1H, quinoline proton), 7.96-7.98 (d, 1H, quinoline proton), 8.24 (s, 1H, C*H*=C(CN)CONH), 8.34-8.37 (d, 1H, quinoline proton), 8.95 (br d, 1H, quinoline proton), 10.42-10.46 (br s, 1H, CON*H*), 12.45-12.58 (br s, 1H, pyrrole N*H*).

**12**	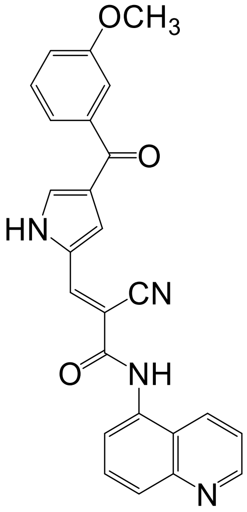	81	>95	423	^{1}H-NMR (DMSO) δ 3.86 (s, 3H, OCH_3), 7.22-7.24 (m, 1H, benzene proton), 7.33 (m, 1H, pyrrole proton), 7.40-7.42 (d, 1H, benzene proton), 7.47-7.51 (t, 1H, benzene proton), 7.57-7.60 (q, 1H, quinoline proton), 7.67-7.69 (d, 1H, quinoline proton), 7.78-7.81 (m, 2H, pyrrole and benzene protons), 7.95-7.99 (m, 2H, quinoline protons), 8.29 (s, 1H, C*H*=C(CN)CONH), 8.37 (d, 1H, quinoline proton), 8.94-8.95 (d, 1H, quinoline proton), 10.43-10.52 (br s, 1H, CON*H*), 12.51-12.70 (br s, 1H, pyrrole N*H*).
**13**	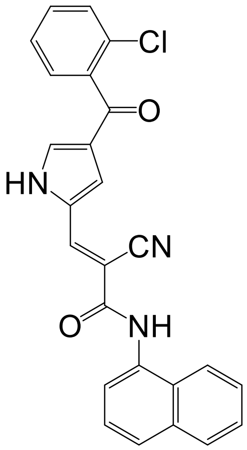	85	>95	426	^{1}H-NMR (DMSO) δ 7.50-7.71 (m, 10H, pyrrole, benzene and naphthalene protons), 7.87-7.89 (d, 1H, naphthalene proton), 7.94-7.99 (m, 2H, naphthalene protons), 8.25 (s, 1H, C*H*=C(CN)CONH), 10.30-10.42 (br s, 1H, CON*H*), 12.56-12.72 (br s, 1H, pyrrole N*H*).
**14**	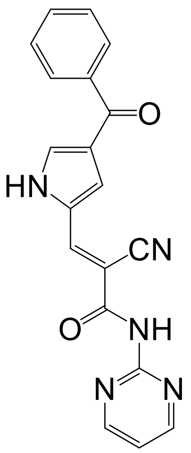	71	>95	344	^{1}H-NMR (DMSO) δ 7.25-7.28 (t, 1H, pyrimidine proton), 7.55-7.58 (t, 2H, pyrrole and benzene protons), 7.64-7.67 (t, 1H, benzene proton), 7.73 (m, 1H, benzene proton), 7.81-7.83 (m, 2H, pyrrole and benzene protons), 7.92 (m, 1H, benzene proton), 8.15 (s, 1H, C*H*=C(CN)CONH), 8.72-8.73 (d, 2H, pyrimidine

					protons) 10.94 (br s, 1H, CON*H*), 12.60 (br s, 1H, pyrrole N*H*).
**15**	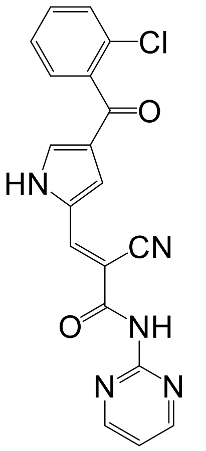	68	>95	378	^{1}H-NMR (DMSO) δ 7.21 (t, 1H, pyrimidine proton), 7.45-7.70 (m, 6H, pyrrole and benzene protons), 8.09 (s, 1H, C*H*=C(CN)CONH), 8.64-8.70 (d, 2H, pyrimidine protons), 10.85-11.03 (br s, 1H, CON*H*), 12.49-12.60 (br s, 1H, pyrrole N*H*).
**16**	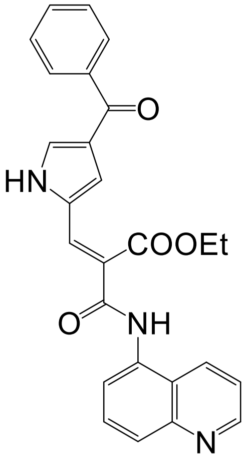	64	>95	440	^{1}H-NMR (DMSO) δ 1.32-1.36 (t, 3H, $OCH_2C\mathit{H_3}$), 4.30-4.33 (q, 2H, $OC\mathit{H_2}CH_3$), 7.10 (m, 1H, pyrrole proton), 7.39-7.43 (t, 2H, quinoline protons), 7.55-7.82 (m, 8H, pyrrole, benzene, quinoline and C*H*=C(CN)CO protons), 7.94-7.97 (d, 1H, quinoline proton), 8.52-8.54 (d, 1H, quinoline proton), 8.94 (d, 1H, quinoline proton), 10.71-10.76 (br s, 1H, CON*H*), 12.26-12.30 (br s, 1H, pyrrole N*H*).

**17**	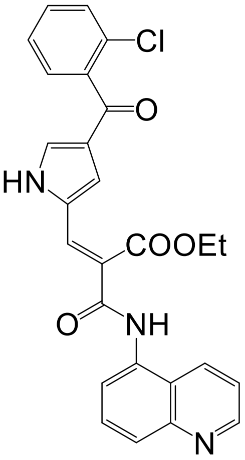	57	>95	474	^{1}H-NMR ($CDCl_3$) δ 1.46-1.48 (t, 3H, OCH_2CH_3), 4.43-4.49 (q, 2H, OCH_2CH_3), 7.21 (t, 1H, pyrrole proton), 7.32-7.53 (m, 5H, benzene and quinoline protons), 7.59 (m, 1H, pyrrole proton), 7.76-7.80 (t, 1H, benzene proton), 8.03 (d, 1H, quinoline proton), 8.17-8.22 (m, 2H, quinoline and C*H*=C(CN)CO protons), 8.46 (d, 1H, quinoline proton), 8.99 (m, 1H, quinoline proton), 11.82-11.85 (br s, 1H, CON*H*), 13.55-13.60 (br s, 1H, pyrrole N*H*).
**18**	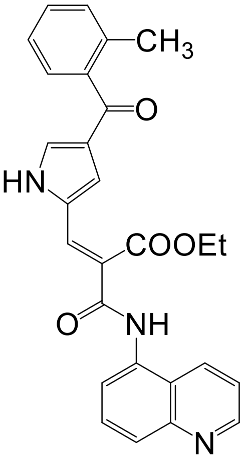	60	>95	454	^{1}H-NMR (DMSO) δ 1.27-1.30 (t, 3H, OCH_2CH_3), 2.17 (s, 3H, CH_3), 4.27 (q, 2H, OCH_2CH_3), 6.88-690 (m, 1H, pyrrole proton), 7.08-7-12 (m, 1H, benzene proton), 7.15-7.19 (m, 2H, benzene protons), 7.24-7.31 (m, 1H, quinoline proton), 7.44-7.54 (m, 3H, pyrrole, quinoline and C*H*=C(CN)CO protons), 7.62-7.64 (m, 1H, benzene proton), 7.72-7.74 (m, 1H quinoline proton), 7.93-7.96 (m, 1H, quinoline proton), 8.47-8.52 (m, 1H, quinoline proton), 8.91 (m, 1H, quinoline proton), 10.61-10.67 (br s, 1H, CON*H*), 12.18-12.36 (br s, 1H, pyrrole N*H*).

**19**	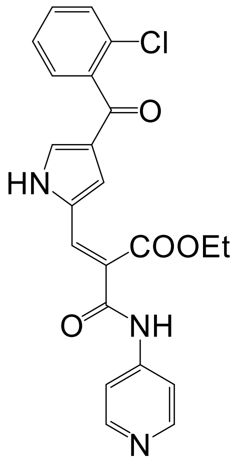	49	>95	424	^{1}H-NMR ($CDCl_3$) δ 1.38 (t, 3H, OCH_2CH_3), 4.31-4.37 (q, 2H, OCH_2CH_3), 7.16- (m, 1H, pyrrole proton) 7.32-7.44 (m, 4H, pyrrole and benzene protons), 7.56-7.58 (d, 2H, pyridine protons), 7.60 (m, 1H, benzene proton), 8.09 (s, 1H, C*H*=C(CN)CONH), 8.51-8.54 (d, 2H, pyridine protons), 11.54 (s, 1H, CON*H*), 13.30-13.34 (br s, 1H, pyrrole N*H*).
**20**	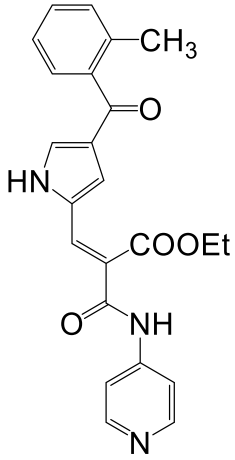	52	>95	404	^{1}H-NMR ($CDCl_3$) δ 1.37 (t, 3H, OCH_2CH_3), 2.37 (s, 3H, CH_3), 4.33-4.37 (q, 2H, OCH_2CH_3), 7.13-7.15 (m, 1H, pyrrole proton), 7.19-7.27 (m, 2H, benzene protons), 7.34-7.41 (m, 2H, pyrrole and benzene protons), 7.54-7.58 (d, 2H, pyridine protons), 7.62-7.64 (m, 1H, benzene proton), 8.07 (s, 1H, C*H*=C(CN)CONH), 8.51-8.53 (d, 2H, pyridine protons), 11.57 (s, 1H, CON*H*), 13.24-13.31 (br s, 1H, pyrrole N*H*).

**21**	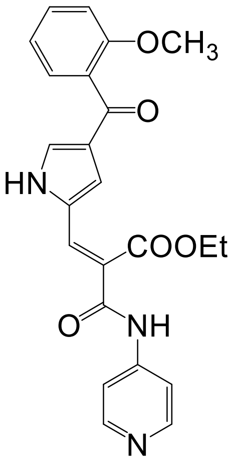	48	>95	420	^{1}H-NMR ($CDCl_3$) δ 1.33-1.41 (t, 3H, OCH$_2$C*H*$_3$), 3.78 (s, 3H, OC*H*$_3$), 4.31-4.37 (q, 2H, OC*H*$_2$CH$_3$), 6.91-7.02 (m, 2H, pyrrole and benzene protons), 7.19-7.23 (m, 1H, benzene proton), 7.36-7.45 (m, 2H, pyrrole and benzene protons), 7.56-7.62 (m, 3H, benzene and pyridine protons), 8.09 (s, 1H, C*H*=C(CN)CONH), 8.50-8.52 (d, 2H, pyridine protons), 11.56 (s, 1H, CON*H*), 13.23 (br s, 1H, pyrrole N*H*).

[a]Elemental analysis was used to determine the purity of compounds that was always >95%. Analytical results were within ± 0.40% of the theoretical values. [b]Low resolution mass spectra were recorded with an API-TOF Mariner by Perspective Biosystem (Stratford, TX, USA); samples were injected by a Harvard pump using a flow rate of 5-10 μL/min with electrospray ionization. [c]^{1}H-NMR spectra were recorded at 400 MHz with a Bruker AC 400 spectrometer, by reporting chemical shifts in δ (ppm) units relative to the internal reference tetramethylsilane.

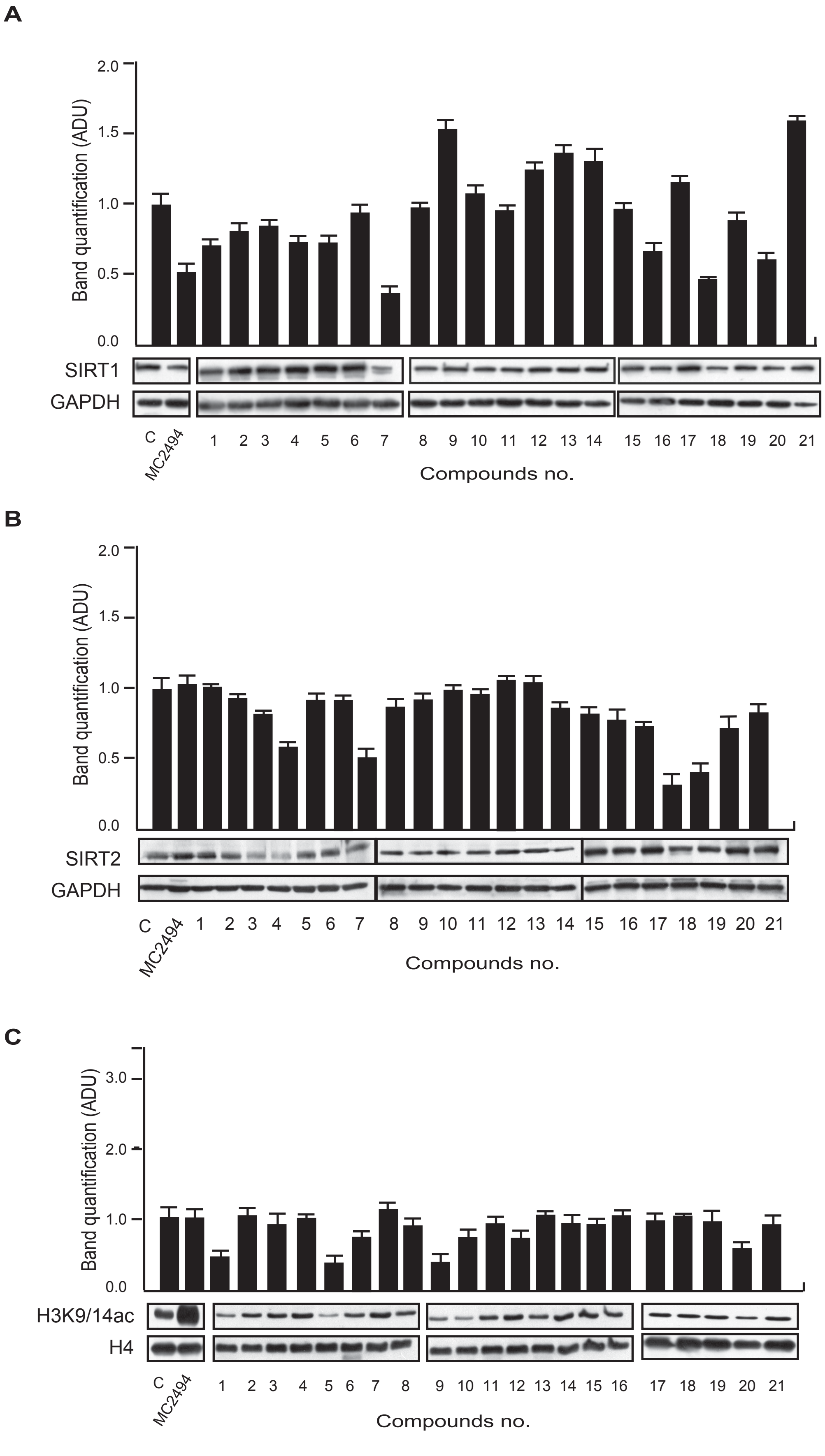
A
B
C
Band quantification (ADU)
SIRT1
GAPDH
SIRT2
H3K9/14ac
H4
C
MC2494
Compounds no.

Supplementary Figure 1. Characterization of novel MC2494-related derivatives.

(A) Western blot analysis of SIRT1. (B) Western blot analysis of SIRT2. (C) Western blot analysis of histone H3 acetylated at lysine K9/14.GAPDH and H4 were used as controls for equal loading. Western blots were normalized through densitometry analysis, performed using the Image J Gel Analysis tool.

Carafa et al. Supplementary Figure 1

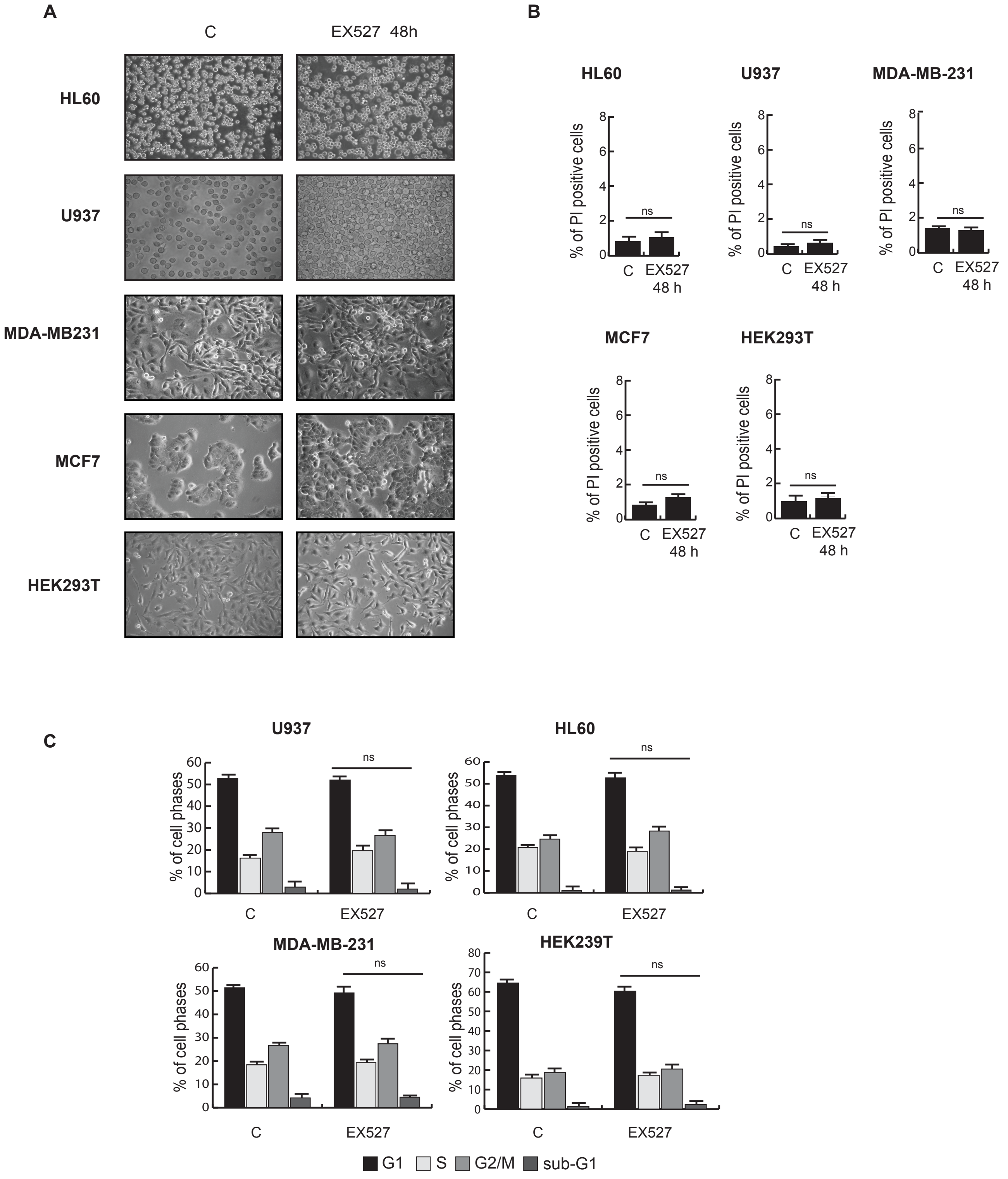

Supplementary Figure 2. EX527: biological characterization.

Cell death analysis performed in HL60, U937, MDA-MB-231, MCF7 and HEK293T, after treatment with EX527 at 5 μM concentration after 48h induction. (A) morphological analysis performed with bright field light microscopy (20X); (B) PI evaluation after treatment with EX527 at 5 μM concentration after 48h induction; (C) Cell cycle evaluation, after treatment with EX527 at 5 μM concentration after 48h induction Graphs show the mean of three independent experiments with error bars indicating standard deviation. Values are mean ± SD of biological triplicates. ＊＊＊＊p-value ≤ 0.0001, ＊＊＊p-value ≤ 0.001, ＊＊p-value ≤ 0.01, ＊p-value ≤ 0.05, ns p-value > 0.05 vs. control cells.

Carafa et al. Supplementary Figure 2

A

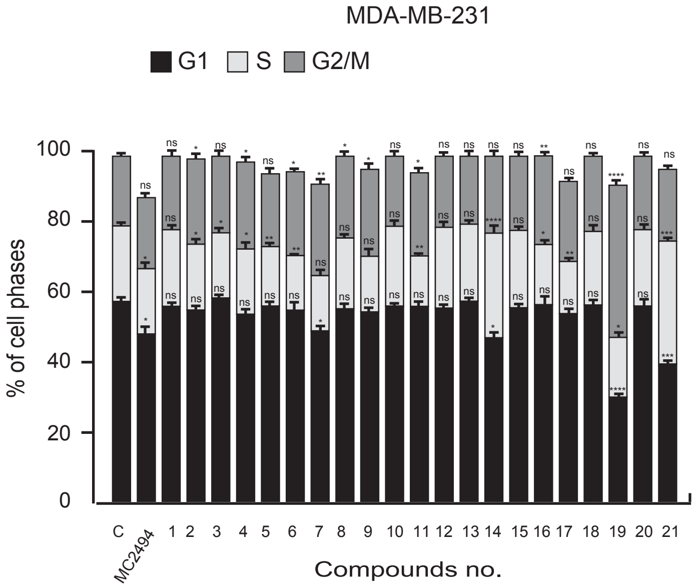

B

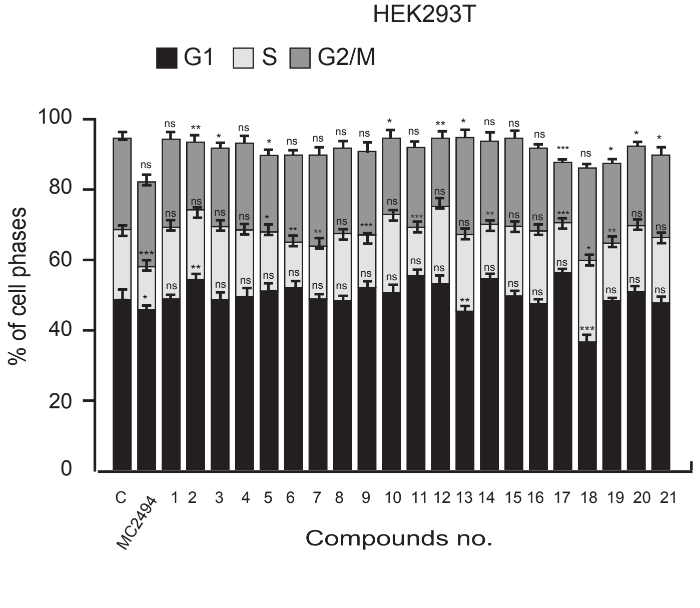

Supplementary Figure 3. MC2494-related derivatives: cell cycle evaluation.
Cell cycle analysis performed with the compounds at 50 μM concentration after 24 h induction.

(A) MDA-MB-231 cells. (B) HEK293T cells. Graphs show the mean of three independent experiments with error bars indicating standard deviation. Values are mean ± SD of biological triplicates. ＊＊＊＊p-value ≤ 0.0001, ＊＊＊p-value ≤ 0.001, ＊＊p-value ≤ 0.01, ＊p-value ≤ 0.05, ns p-value > 0.05 vs. control cells.

Carafa et al. Supplementary Figure 3

A

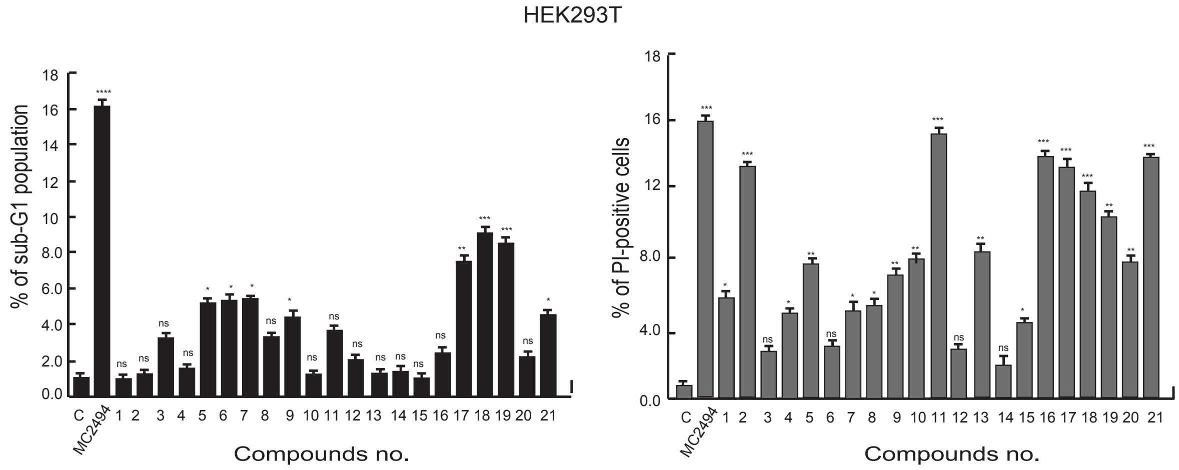

B

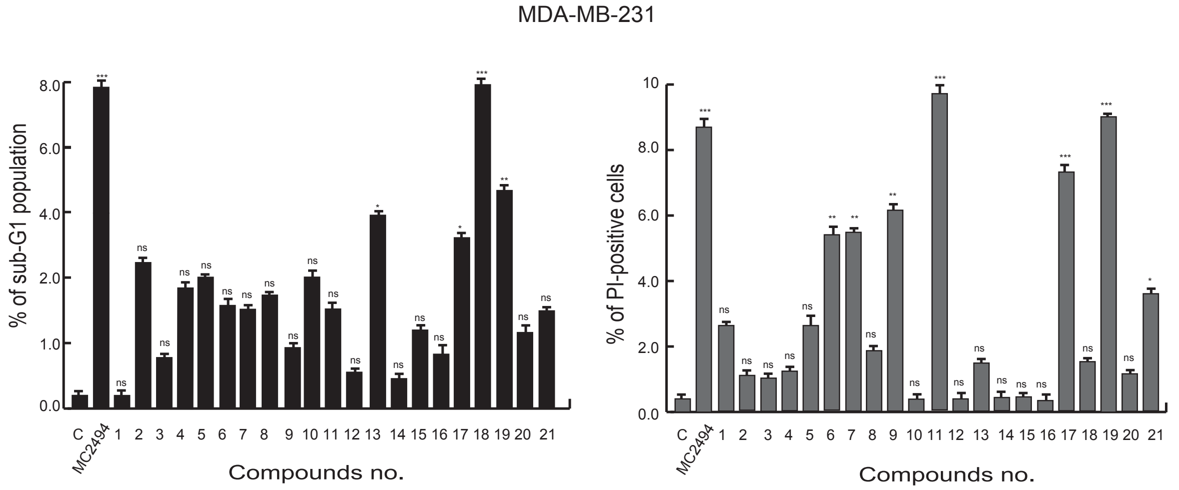

C

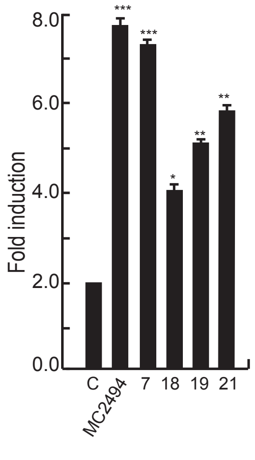

Supplementary Figure 4. MC2494-related derivatives: cell death evaluation.

Cell death analysis, performed with the compounds at 50 μM concentration after 24 h induction.
(A) Cell death evaluation performed in HEK293T; sub-G1 evaluation (*Left panel*), PI evaluation (*Right panel*). (B) Cell death evaluation performed in MDA-MB-231; sub-G1 evaluation (*Left panel*), PI evaluation (*Right panel*). (C) Caspase 3/7 performed in U937cellswith the compounds.

Graphs show the mean of three independent experiments with error bars indicating standard deviation.
Values are mean ± SD of biological triplicates. ∗∗∗∗p-value ≤ 0.0001, ∗∗∗p-value ≤ 0.001, ∗∗p-value ≤ 0.01, ∗p-value ≤ 0.05, ns p-value > 0.05 vs. control cells.

A

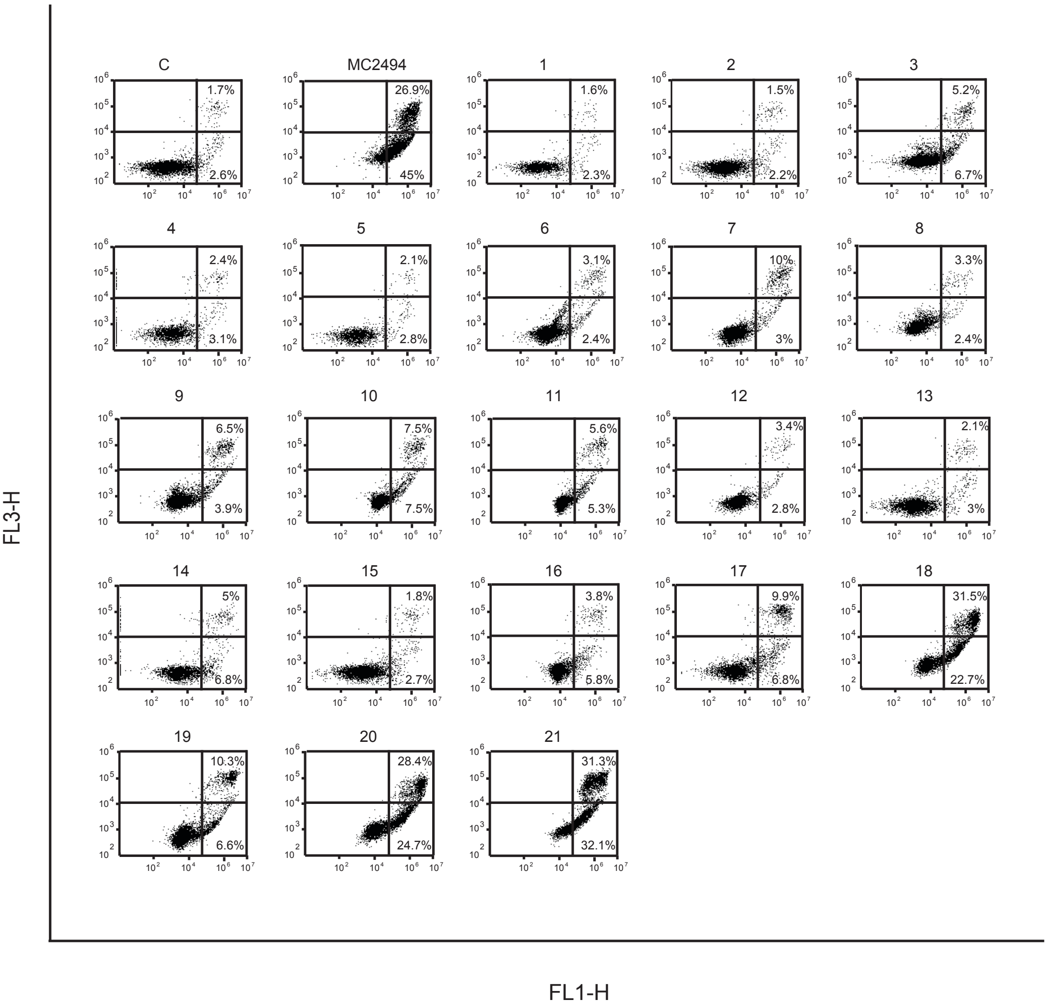

B

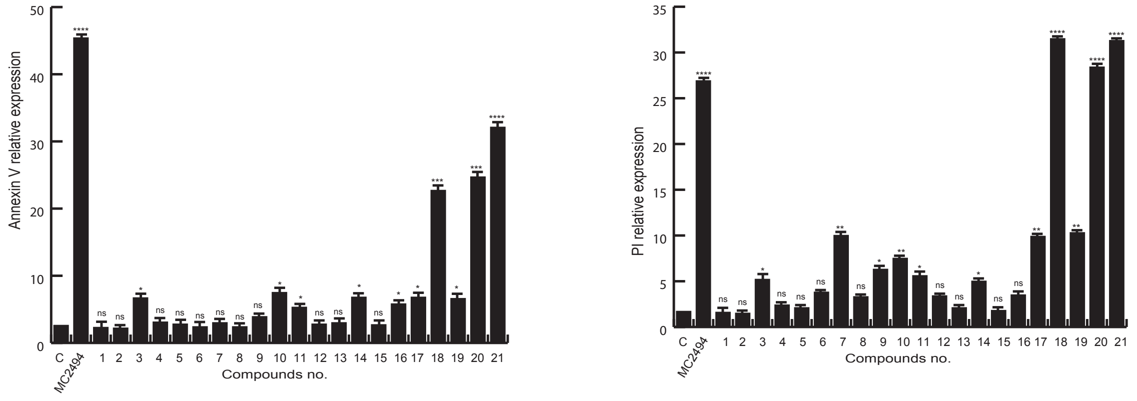

Supplementary Figure 5. MC2494-related derivatives: apoptosis evaluation.
Annexin V/PI assays, were performed in U937cellswith the compounds at 50 μM concentration after 24 h induction.
(A) Density pltots of Annexin V/PI evaluation performed with indicated compounds.(B) Annessin V/PI evaluation. *left panel:*Annexin V relative expresssion; *right panel:* PI relative expression.Graphs show the mean of three independent experiments with error bars indicating standard deviation.
Values are mean ± SD of biological triplicates. ∗∗∗∗p-value ≤ 0.0001, ∗∗∗p-value ≤ 0.001, ∗∗p-value ≤ 0.01, ∗p-value ≤ 0.05, ns p-value > 0.05 vs. control cells.

A

MCF7 cells

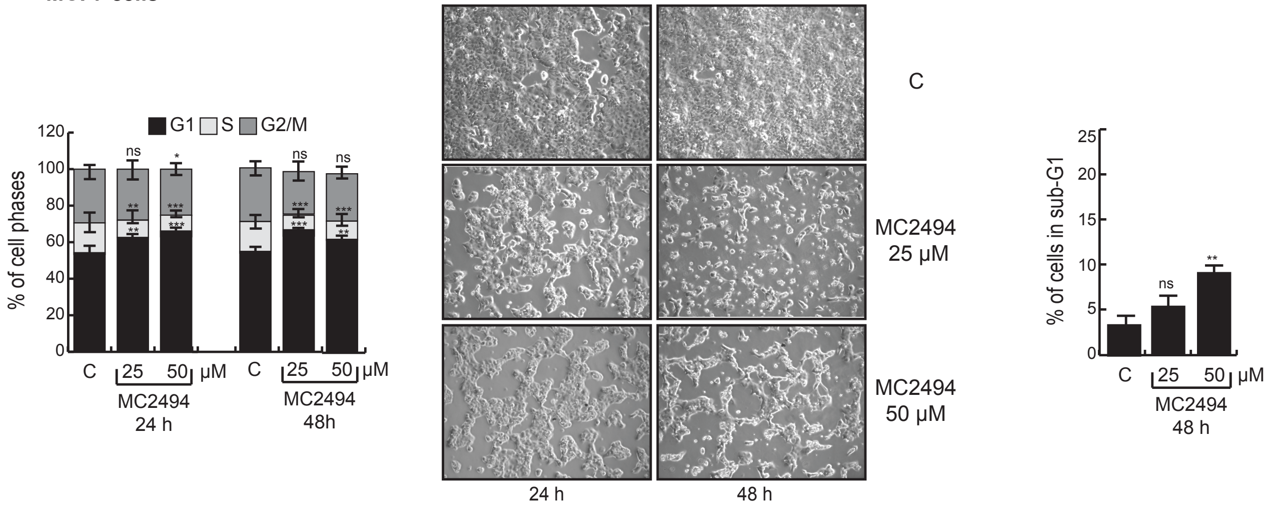

B

MDA-MB-231 cells

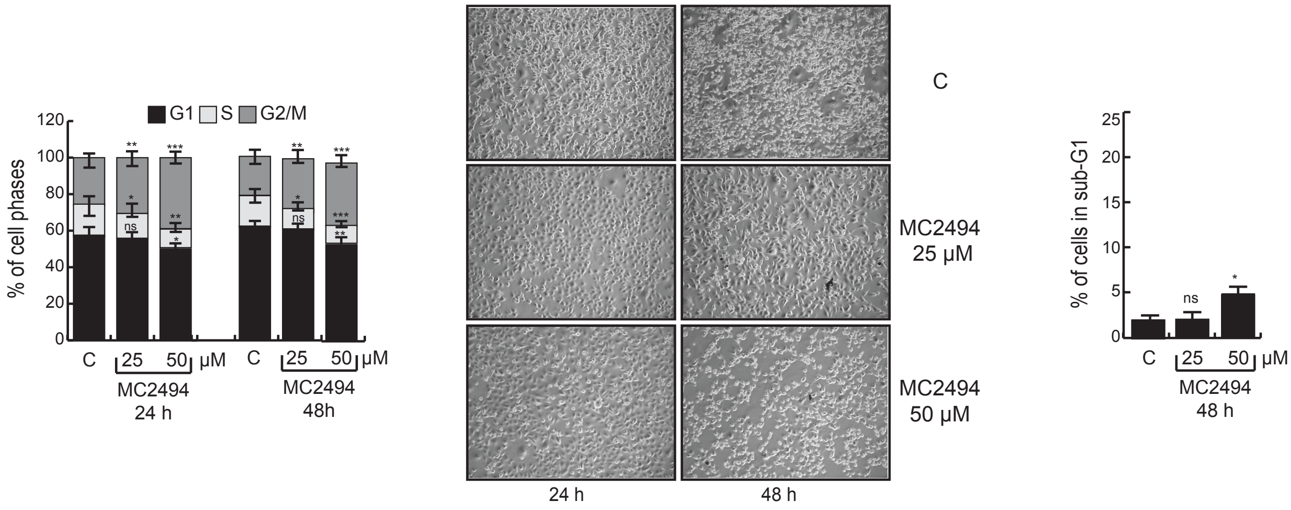

Supplementary Figure 6. MC2494 affect proliferation in breast cancer MCF7 and MDA-MB-231 cell lines.

Both cell lines were treated with MC2494 for 24 h and 48 h at two different concentrations (25 μM and 50 μM).
Cell cycle analysis (left panels), morphological analysis performed with bright field light microscopy (20X) (middle panels), sub-G1 analysis (right panels).
(A) Experiments performed with MCF7 cells. (B) Experiments performed with MDA-MB-231 cells.
Graphs show the mean of at least two independent experiments with error bars indicating standard deviation. Values are mean ± SD of biological triplicates. ＊＊＊＊p-value ≤ 0.0001, ＊＊＊p-value ≤ 0.001, ＊＊p-value ≤ 0.01, ＊p-value ≤ 0.05, ns p-value > 0.05 vs. control cells.

A

MDA 453 cells

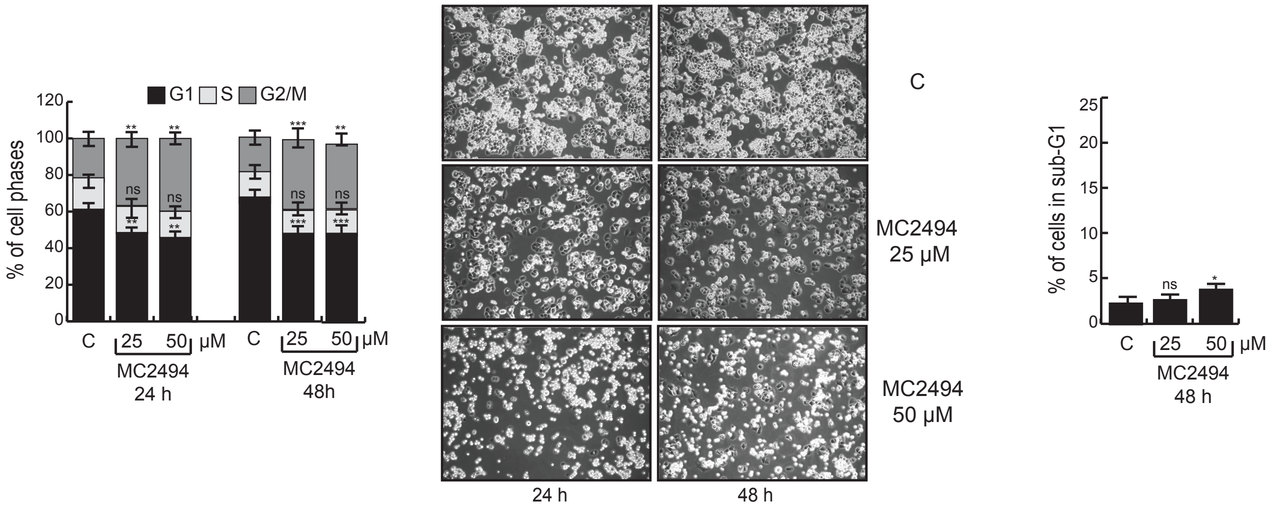

B

T47D cells

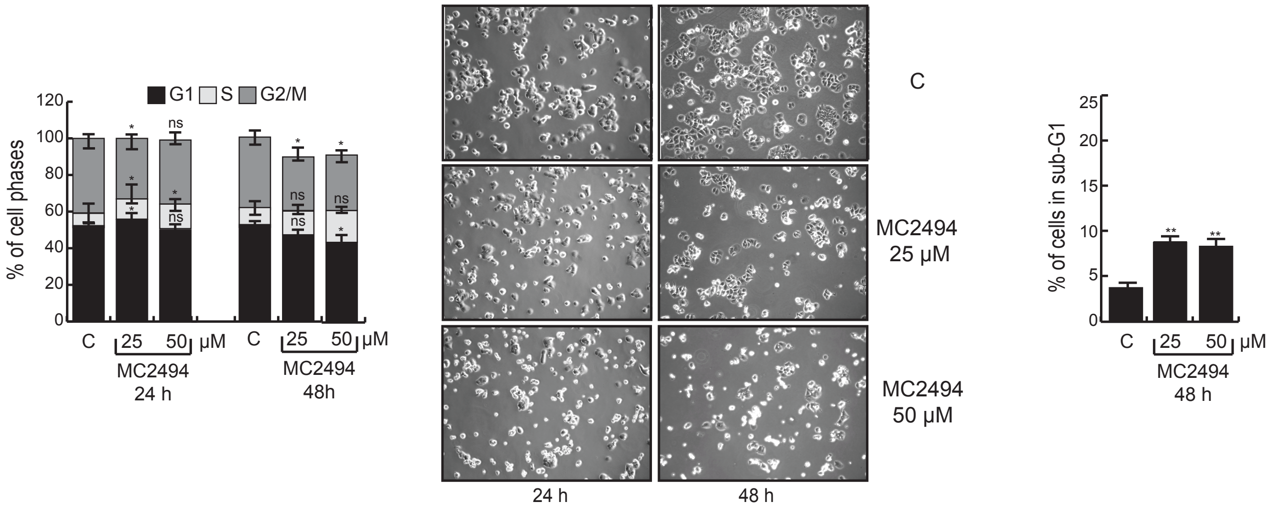

Supplementary Figure 7. MC2494 affect proliferation in breast cancer MDA 453 and T47D cell lines.

Both cell lines were treated with MC2494 for 24 h and 48 h at two different concentrations (25 µM and 50 µM).
Cell cycle analysis (left panels), morphological analysis performed with bright field light microscopy (20X) (middle panels), sub-G1 analysis (right panels).
(A) Experiments performed with MDA 453 cells. (B) Experiments performed with T47D cells.
Graphs show the mean of at least two independent experiments with error bars indicating standard deviation. Values are mean ± SD of biological triplicates. ＊＊＊＊p-value ≤ 0.0001, ＊＊＊p-value ≤ 0.001, ＊＊p-value ≤ 0.01, ＊p-value ≤ 0.05, ns p-value > 0.05 vs. control cells.

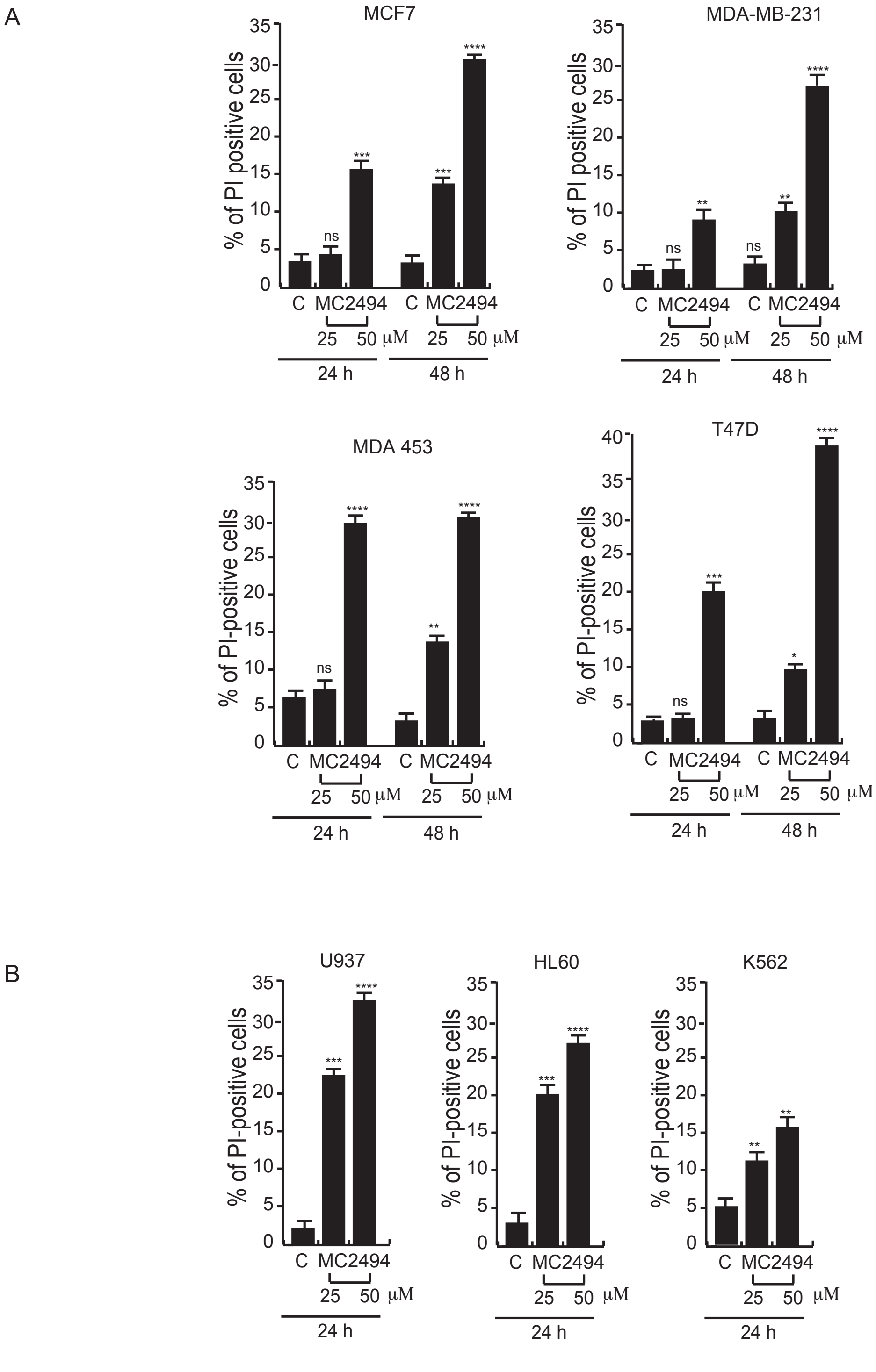

Supplementary Figure 8. MC2494 induces cell death.

(A) PI evaluation in breast cancer cells performed with MC2494 at 25 μM and 50 μM concentration after 24 h and 48 h induction.
(B) PI evaluation in leukemia cells performed with MC2494 at 25 μM and 50 μM concentration after 24 h induction.
Graphs show the mean of three independent experiments with error bars indicating standard deviation. Values are mean ± SD of biological triplicates. ****p-value ≤ 0.0001, ***p-value ≤ 0.001, **p-value ≤ 0.01, *p-value ≤ 0.05, ns p-value > 0.05 vs. control cells.

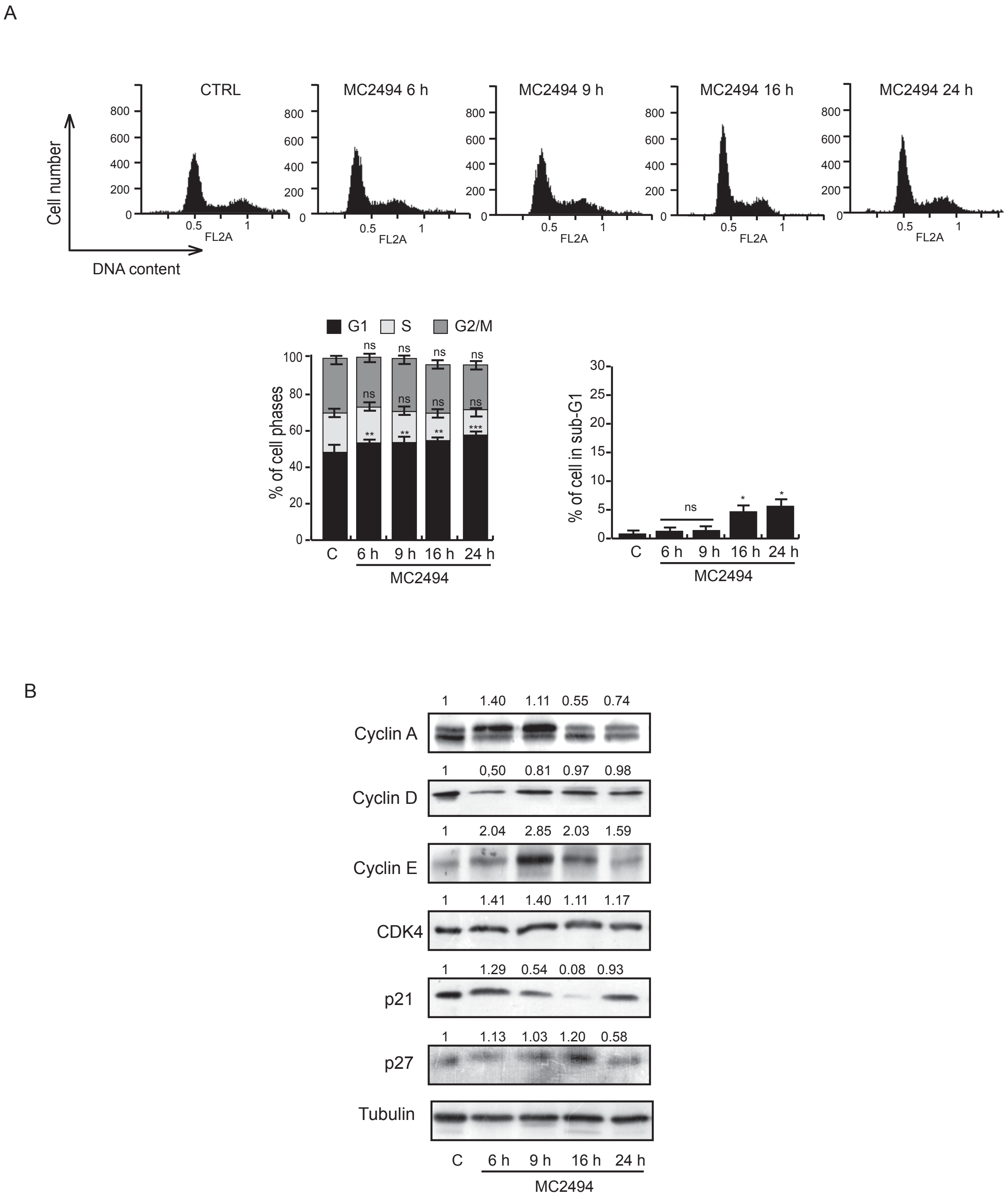

Supplementary Figure 9. MC2494: effect on cell cycle regulation.

MCF7 cells were treated with MC2494 for indicated times at 50 µM.
(A) Top: Cell cycle distribution graphs; Bottom: cell cycle and sub-G1 analysis. (B)Western blot analysis of indicated cell cycle regulatory proteins. GAPDH was used as control for equal loading. Numbers upon each Western blot are the results of densitometry analysis performed using Image J Analysis Tool .Graphs show the mean of three independent experiments with error bars indicating standard deviation. Values are mean ± SD of biological triplicates. ＊＊＊＊p-value ≤ 0.0001, ＊＊＊p-value ≤ 0.001, ＊＊p-value ≤ 0.01, ＊p-value ≤ 0.05, ns p-value > 0.05 vs. control cells.
